# Genomic characterization of liver metastases from colorectal cancer patients

**DOI:** 10.18632/oncotarget.12140

**Published:** 2016-09-20

**Authors:** José María Sayagués, Luís Antonio Corchete, María Laura Gutiérrez, Maria Eugenia Sarasquete, María del Mar Abad, Oscar Bengoechea, Encarna Fermiñán, María Fernanda Anduaga, Sofia del Carmen, Manuel Iglesias, Carmen Esteban, María Angoso, Jose Antonio Alcazar, Jacinto García, Alberto Orfao, Luís Muñoz-Bellvis

**Affiliations:** ^1^ Cytometry Service-NUCLEUS, Department of Medicine, Cancer Research Center, IBMCC-CSIC/USAL and IBSAL, University of Salamanca, Salamanca, Spain; ^2^ Cancer Research Center and Service of Hematology, University Hospital of Salamanca, Salamanca, Spain; ^3^ Department of Pathology, University Hospital of Salamanca, Salamanca, Spain; ^4^ Genomics Unit, Cancer Research Center, IBMCC-CSIC/USAL, Salamanca, Spain; ^5^ Service of General and Gastrointestinal Surgery and IBSAL, University Hospital of Salamanca, Salamanca, Spain

**Keywords:** GEP, colorectal cancer

## Abstract

Metastatic dissemination is the most frequent cause of death of sporadic colorectal cancer (sCRC) patients. Genomic abnormalities which are potentially characteristic of such advanced stages of the disease are complex and so far, they have been poorly described and only partially understood. We evaluated the molecular heterogeneity of sCRC tumors based on simultaneous assessment of the overall GEP of both coding mRNA and non-coding RNA genes in primary sCRC tumor samples from 23 consecutive patients and their paired liver metastases. Liver metastases from the sCRC patients analyzed, systematically showed deregulated transcripts of those genes identified as also deregulated in their paired primary colorectal carcinomas. However, some transcripts were found to be specifically deregulated in liver metastases (*vs*. non-tumoral colorectal tissues) while expressed at normal levels in their primary tumors, reflecting either an increased genomic instability of metastatic cells or theiradaption to the liver microenvironment. Newly deregulated metastatic transcripts included overexpression of APOA1, HRG, UGT2B4, RBP4 and ADH4 mRNAS and the miR-3180-3p, miR-3197, miR-3178, miR-4793 and miR-4440 miRNAs, together with decreased expression of the IGKV1-39, IGKC, IGKV1-27, FABP4 and MYLK mRNAS and the miR-363, miR-1, miR-143, miR-27b and miR-28-5p miRNAs. Canonical pathways found to be specifically deregulated in liver metastatic samples included multiple genes related with intercellular adhesion and the metastatic processes (e.g., IGF1R, PIK3CA, PTEN and EGFR), endocytosis (e.g., the PDGFRA, SMAD2, ERBB3, PML and FGFR2), and the cell cycle (e.g., SMAD2, CCND2, E2F5 and MYC). Our results also highlighted the activation of genes associated with the TGFβ signaling pathway, -e.g. RHOA, SMAD2, SMAD4, SMAD5, SMAD6, BMPR1A, SMAD7 and MYC-, which thereby emerge as candidate genes to play an important role in CRC tumor metastasis.

## INTRODUCTION

Occurrence of distant metastases is the main cause of sporadic colorectal cancer (sCRC) death, and the liver is the most common site for metastatic spread of the primary tumor [1, 2]. Metastasis is a complex multi-step process leading to the accumulation of genomic alterations in single tumor cells over the lifetime of a tumor from a benign lesion to invasive and metastatic spreading states leading to patient death [3, 4]. Genomic abnormalities which are potentially characteristic of such advanced stages of the disease are complex and so far, they have been poorly described and only partially understood. This relates to the fact that most genomic studies performed in colorectal cancer have focused on primary tumors, particularly in stage II disease, at diagnosis; in contrast, few studies have compared the deregulated transcripts of primary versus paired metastatic samples. Despite this, multiple mRNAs and miRNAS found to be expressed in primary tumors have been associated with metastatic colorectal carcinoma. Among others, these include mRNA of PTEN/PI3K [5], EGFR [6], TGFβ [7], and TP53 [8], as well as the metastatic CRC-associated miRNAs, miR-31 [9], miR-503 [10] and miR-133a [11].

Microarray analysis allows simultaneous investigation of several thousands of cancer-related and cancer-specific genes [12], such gene expression profiling (GEP) being highlighted as a potential tool to identify biomarkers for better prognostication and treatment of different types of cancer, including sCRC [13]. Thus, microarray data might provide significant insight into the biological differences between patients within a given cancer type with good- and poor-prognosis, at the same time it might be used as a screening tool for the identification of molecules to be targeted by existing or future (individualized) therapies. In recent years, several GEP have been identified as predictors for CRC stage II patient outcome. These include those being tested with the Oncotype DX^®^ Colon Cancer test (Genomic Health, Inc., Redwood City, CA) [14] and Coloprint^®^ (Agendia, Inc., Irvine, CA) gene chips, among other GEP platforms [13]. However, the molecular mechanisms underlying the association of such genomic profiles with metastatic colorectal carcinoma remain largely unknown.

Here we evaluate the molecular heterogeneity of sCRC tumors based on simultaneous assessment of the overall GEP for both coding mRNA and non-coding RNA genes -including miRNA, small nucleolar and large intergenic RNAs- in primary sCRC tumor samples from 23 consecutive patients and their paired liver metastases *vs.* non-tumoral tissues (*n* = 9). Overall, our results define a common GEP for all metastatic sCRC, which confirms and extends on previous observations [15] revealing new (specific) mRNA and miRNA signatures to be (potential) protective biomarker signatures for distant-disease.

## RESULTS

### Overall transcriptional profile of metastatic sCRC

**Figure 1:**
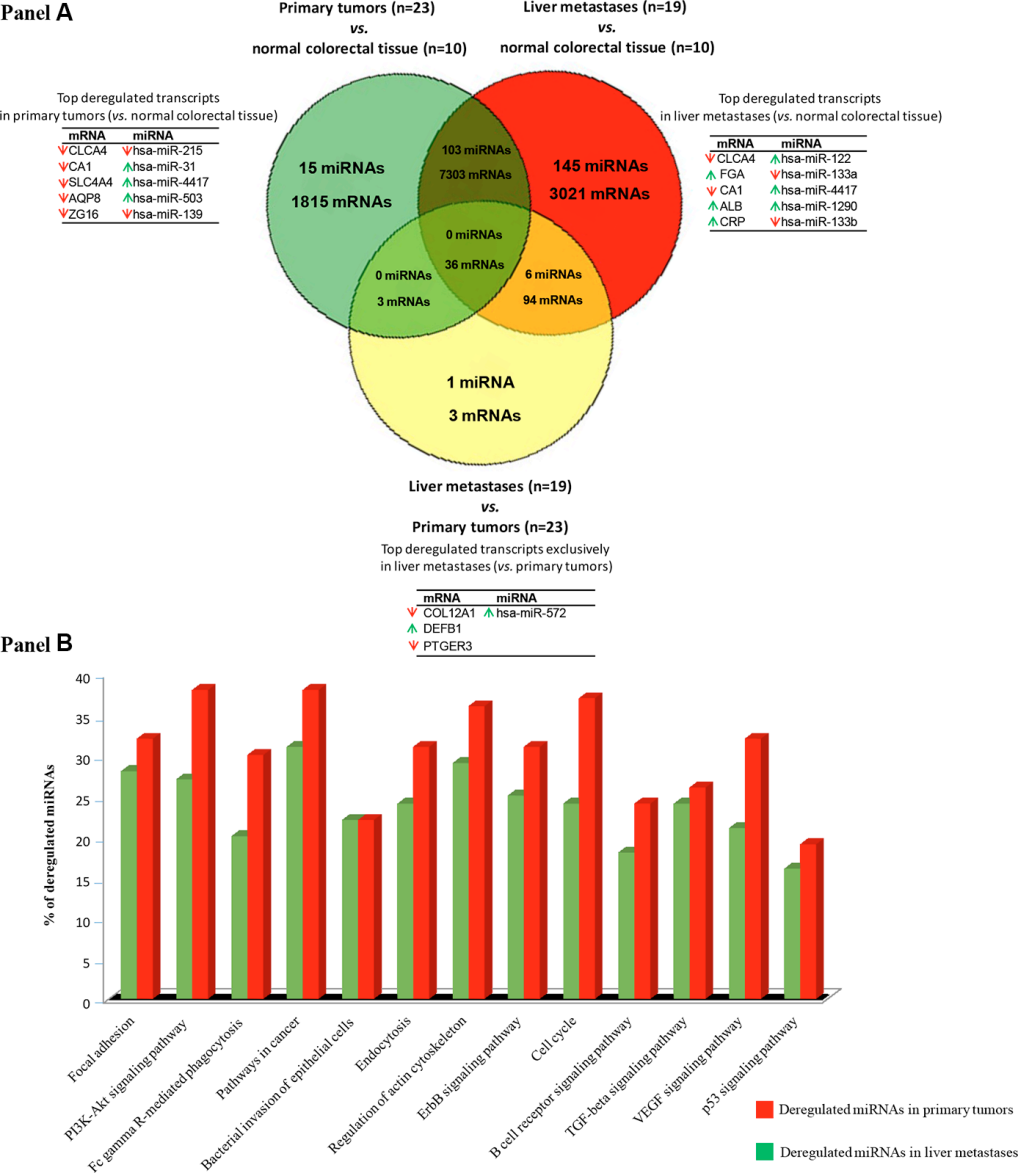
Gene expression profiles (GEP) of primary vs. metastatic colorectal cancer (Panel **A**) Venn diagram representing mRNA and miRNA differentially expressed between the different types of samples analyzed: primary sporadic colorectal tumors *vs.* normal colorectal tissue, colorectal liver metastases *vs.* normal colorectal tissue and primary sporadic colorectal tumors *vs.* their paired liver metastases (*q*-values < 0.01). (Panel **B**) Most represented canonical pathways involved in metastatic colorectal tumors as defined by their GEP of both coding and non-coding RNAs (*q* < 0.01).

**Figure 2:**
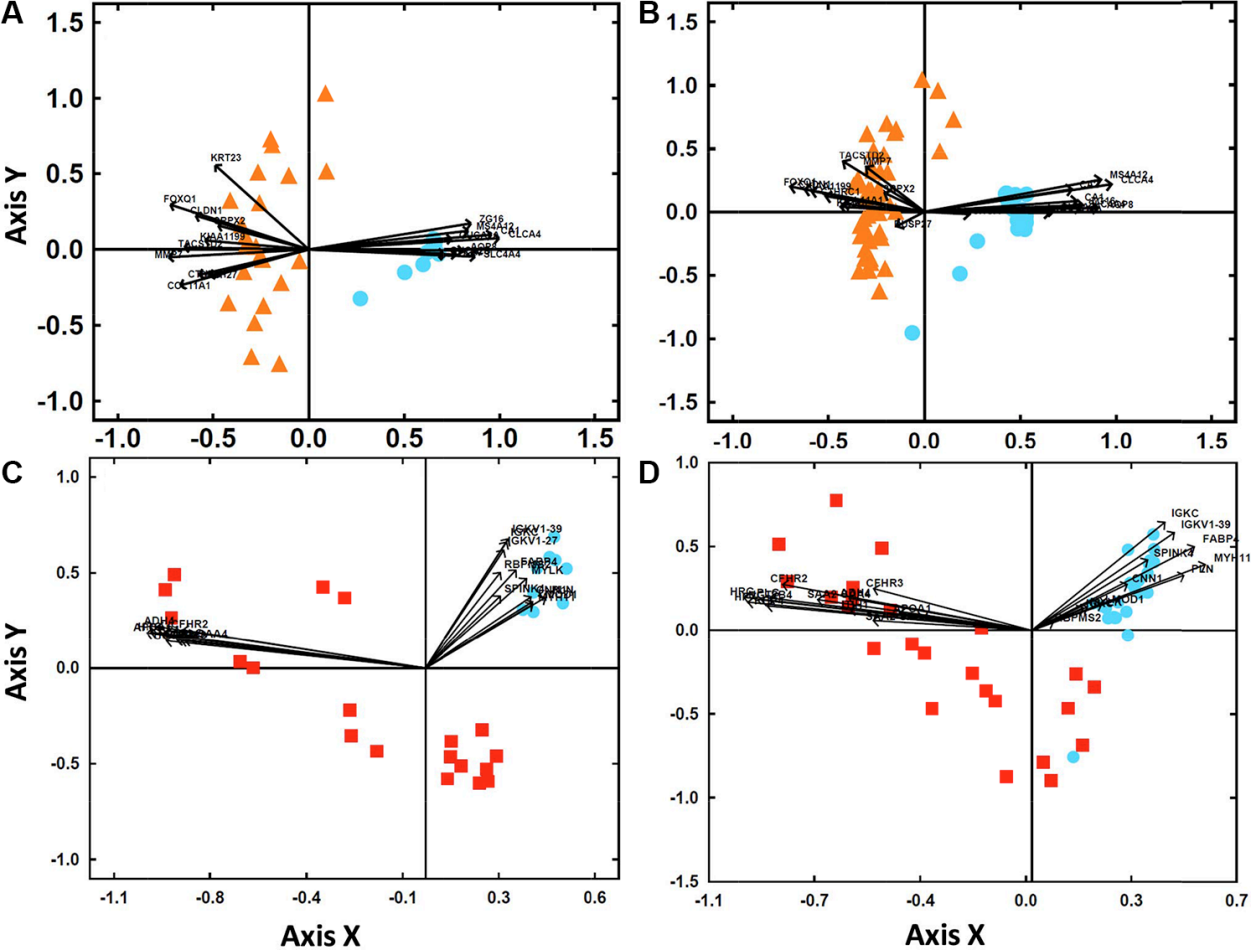
Classification of sCRC tumors vs non-tumoral colorectal tissues based on the gene expression profile (GEP) of those transcripts more strongly deregulated in primary tumors and their liver metastases (Panel **A**) Biplot analysis of 23 primary colorectal tumors (orange triangles) *vs.* 10 non-tumoral colorectal tissues (blue circles) from our sCRC patient series. (Panel **B**) Biplot analysis of 47 primary colorectal tumors (orange triangles) *vs.* 25 non-tumoral colorectal tissue samples (blue circles) from an independent external validation dataset (GEO database, accession number GSE21510). (Panel **C**) Biplot analysis of 19 liver metastases (red squares) *vs.* 10 non-tumoral colorectal tissues (blue circles) from our sCRC patient series. (Panel **D**) Biplot analysis of 24 liver metastases (red squares) *vs.* 23 non-tumoral colorectal tissues (blue circles) from an independent external validation dataset (GEO database, accession number GSE35834).

Venn diagram analysis showed a total of 9,157 mRNA (3,744 down- and 5,413 up-regulated genes) and 118 miRNA genes (60 down- and 58 up-regulated) to be deregulated in primary tumors (*vs.* non-tumoral colorectal tissue) and 10,454 mRNA (4,777 down- and 5,677 up-regulated) and 254 miRNA genes (68 down- and 186 up-regulated) to be altered in liver metastases (*vs.* non-tumoral colorectal tissue). Overall, primary tumors and their paired liver metastases shared a total of 7,339 mRNA (3,297 down- and 4,042 up-regulated) and 109 miRNA (53 down- and 56 up-regulated) deregulated genes (Figure 1). Interestingly, one quarter of all mRNA (1,815/9,157; 20%) and miRNA transcripts (15/118; 13%) was exclusively found in primary tumors while one third of all differentially expressed mRNA transcripts (3,021/10,454; 29%) and the majority miRNA transcripts (145/254; 57%) were found to be deregulated only in liver metastases. Those genes most strongly overexpressed in primary tumors included the FOXQ1, MMP7, CLDN1, TACSTD2 and COL11A1 mRNAs and the miR-31, miR-4417, miR-503, miR-3647 and miR-592 miRNA transcripts; in turn, the CLCA4, CA1, SLC4A4, AQP8, and ZG16 mRNAs together with the miR-215, miR-139, miR-133a, miR-378c and miR-378d miRNAs were those genes showing the strongest down-regulated levels across all primary tumor samples analyzed (Table 1). Of note, those mRNAs and miRNAs transcripts differentially expressed in primary tumors vs. non-tumoral colorectal tissues, allowed for clear cut discrimination between both types of samples (Figure 2).

**Table 1:** Top mRNAs and miRNAs up- and down-regulated in primary sporadic colorectal tumors (*n* = 23) *vs*. non-tumoral colorectal tissues (*n* = 10)

Gene Name	Gene ID	Fold Change T vs. Non-T	Chr. band	Start (bp)	Stop (bp)	Strand	Transcript description
**Up-regulated mRNA transcripts**							
FOXQ1	ENSG00000164379	44.1	6p25	1312440	1314758	+	protein-coding
MMP7	ENSG00000137673	31.1	11q21	102520508	102530753	−	protein-coding
CLDN1	ENSG00000163347	26.2	3q28	190305701	190322475	−	protein-coding
TACSTD2	ENSG00000184292	22.9	1p32	58575423	58577773	−	protein-coding
COL11A1	ENSG00000060718	21.4	1p21	102876467	103108496	−	protein-coding
KIAA1199	ENSG00000103888	17.1	15q24	80779343	80951776	+	protein-coding
CTHRC1	ENSG00000164932	14.7	8q22	103371515	103383005	+	protein-coding
DUSP27	ENSG00000198842	14.4	1q24	167094045	167129165	+	protein-coding
KRT23	ENSG00000108244	13.8	17q21	40922696	40937643	−	protein-coding
SRPX2	ENSG00000102359	12.3	Xq22	100644166	100671299	+	protein-coding
NFE2L3	ENSG00000050344	10.8	7p15	26152227	26187137	+	protein-coding
**Up-regulated miRNA transcripts**							
hsa-miR-31	ENSG00000199177	10.3	9p21	21512114	21512184	−	hsa-miR
hsa-miR-4417	ENSG00000264341	8.2	1p36	5624131	5624203	+	hsa-miR
hsa-miR-503	ENSG00000208005	8.1	Xq26	133680358	133680428	−	hsa-miR
hsa-miR-1290	ENSG00000221662	5.9	1p34	18897071	18897148	−	hsa-miR
hsa-miR-3687	ENSG00000264063	5.5	21p11	9826203	9826263	+	hsa-miR
hsa-miR-592	ENSG00000207692	4.9	7q31	126698142	126698238	−	hsa-miR
hsa-miR-183	ENSG00000207691	4.6	7q32	129774905	129775014	−	hsa-miR
hsa-miR-224	ENSG00000207621	4.5	Xq28	151958578	151958658	−	hsa-miR
hsa-miR-1246	ENSG00000207584	4.2	2q31	176600980	176601052	−	hsa-miR
hsa-miR-21	ENSG00000199004	3.9	17q23	59841266	59841337	+	hsa-miR
hsa-miR-424	ENSG00000223749	3.8	Xq26	133677367	133680741	−	hsa-miR
**Down-regulated mRNA transcripts**							
CLCA4	ENSG00000016602	−134.1	1p22	87012759	87046437	+	protein-coding
CA1	ENSG00000133742	−85.4	8q21	86239837	86291243	−	protein-coding
SLC4A4	ENSG00000080493	−60.8	4q21	71092756	71572087	+	protein-coding
AQP8	ENSG00000103375	−51.8	16p12	25227052	25240261	+	protein-coding
ZG16	ENSG00000174992	−45.6	16p11	29778240	29782973	+	protein-coding
MS4A12	ENSG00000071203	−43.8	11q12	60260251	60274903	+	protein-coding
CA2	ENSG00000104267	−37.2	8q21	85463852	85481493	+	protein-coding
GUCA2B	ENSG00000044012	−35.7	1p34	42619092	42621495	+	protein-coding
CHGA	ENSG00000100604	−35.2	14q32	93389425	93401638	+	protein-coding
GUCA2A	ENSG00000197273	−35.2	1p34	42628362	42630395	−	protein-coding
CLCA1	ENSG00000016490	−34.8	1p22	86934051	86965977	+	protein-coding
SLC26A3	ENSG00000091138	−33.7	7q31	107405912	107443678	−	protein-coding
UGT2B17	ENSG00000197888	−31	4q13	69402902	69434245	−	protein-coding
**Down-regulated miRNA transcripts**							
hsa-miR-215	ENSG00000207590	−11	1q41	220117853	220117962	+	hsa-miR
hsa-miR-139-5p	ENSG00000272036	−8	11q13	72615063	72615130	−	hsa-miR
hsa-miR-133a	ENSG00000207764	−7.1	20q13	61162119	61162220	+	hsa-miR
hsa-miR-378c	ENSG00000264803	−7	10q26	130962588	130962668	−	hsa-miR
hsa-miR-378d	ENSG00000263631	−6.7	4p16	5923275	5923328	−	hsa-miR
hsa-miR-422a	ENSG00000199156	−6.6	15q22	63870930	63871019	−	hsa-miR
hsa-miR-375	ENSG00000198973	−6.4	2q35	219866362	219866431	−	hsa-miR
hsa-miR-378f	ENSG00000264926	−6.3	1p36	23929070	23,929,147	+	hsa-miR
hsa-miR-378i	ENSG00000263463	−6.1	22q13	41923222	41923297	−	hsa-miR
hsa-miR-378g	ENSG00000263526	−5.6	1p21	94745860	94745900	−	hsa-miR
hsa-miR-133b	ENSG00000199080	−5.4	6p22	52148923	52149041	+	hsa-miR
hsa-miR-138	ENSG00000207954	−4.5	3p21	44114212	44114310	+	hsa-miR
hsa-miR-378e	ENSG00000263831	−4.2	5q35	169455492	169455570	+	hsa-miR
hsa-miR-143	ENSG00000208035	−3.7	5q32	148808481	148808586	+	hsa-miR

*q*-values < .01; T: tumoral samples; Non-T: non-tumoral samples; hsa-miR: human micro-RNA.

Overall, liver metastases from the 19 colorectal cancer patients analyzed systematically showed deregulated transcripts of those genes identified as being also deregulated in their paired primary colorectal carcinomas (Table 2). Thus, both the primary tumors and their paired liver metastases showed overexpression of FOXQ1, MMP7, CLDN1 and TACSTD2 mRNAS and the miR-4417, miR-503, miR-1290, miR-3687, miR-183, miR-224 and miR-1246 miRNAs, together with down-regulated levels of the CLCA4, CA1, AQP8, ZG16, GUCA2B and SLC26A3 mRNAS and the miR-215, miR-133a, miR-375, miR-133b and miR-138 miRNAs. Interestingly, most of these genes with highly deregulated expression in both primary tumors and their corresponding liver metastases have been previously found to be altered/involved in colorectal cancer (e.g., FOXQ1, MMP7, TACSTD2 CLCA4, CA1, AQP8, ZG16, GUCA2B and SLC26A3) and/or they have been identified as genes that are relevant to the metastatic process (e.g., FOXQ1, MMP7, TACSTD2 CLCA4, CA1, AQP8 and SLC26A3); similarly the miRNAs, miR-503, miR-3687, miR-215, miR-133a, miR-375, miR-183, miR-1290, miR-224, miR-1246, have also been reported to be typically altered in CRC. Despite the similarities observed between the GEP of primary and metastatic tumors, some transcripts were found to be specifically deregulated in liver metastases while expressed at normal levels in their paired primary tumors, reflecting either an increased genomic instability of metastatic cells or their adaption to the liver microenvironment (Table 3). Newly deregulated metastatic transcripts included overexpression of APOA1, HRG, UGT2B4, RBP4 and ADH4 mRNAS and the miR-3180-3p, miR-3197, miR-3178, miR-4793 and miR-4440 miRNAs, together with decreased expression of the IGKV1-39, IGKC, IGKV1-27, FABP4 and MYLK mRNAS and the miR-363, miR-1, miR-143, miR-27b and miR-28-5p miRNAs. Furthermore, once the 19 paired liver metastasis and primary tumor samples were specifically compared (paired analysis) 52 mRNAS (14 down- and 38 up-regulated genes) and only two over-expressed miRNAs were observed (miR-122 and miR-4322; Supplementary Table 2), these including the most differentially expressed mRNAs and miRNAs identified with the unpaired comparison of all samples. Of note, among the regulated miRNAs in both the paired and unpaired comparisons was miR-122, a miRNA which is highly expressed in the liver, wich could potentially be due to the presence of normal liver tissue in the metastasis samples. In order to determine the degree of contamination by residual liver cells in metastatic tissues, we analyzed the expression levels of 41 liver-associated genes, as selected from the Tissue-specific Gene Expression and Regulation database (TIGER) plus miR-122 in normal liver (*n* = 5), CRC liver metastases (*n* = 19), normal colorectal mucosa (n=9) and primary CRC tumors (*n* = 23). All 41 liver-specific genes showed similar expression levels in CRC liver metastases, normal colorectal mucosa and primary CRC tumors, while they were highly expressed in the normal liver samples (Supplementary Figure 1). In contrast, miR122 was highly expressed in the liver and CRC liver metastases, whereas it showed lower expression levels in normal mucosa and primary CRC tumors, suggesting that over-expression of miR122 in liver metastatic tissues was not specifically due to residual liver cell-associated background in the metastatic CRC tissues.

**Table 2:** Top mRNAs and miRNAs up- and down-regulated in colorectal liver metastases (*n* = 19) *vs*. non-tumoral colorectal tissues (*n* = 10)

Gene Name	Gene ID	Fold Change T vs. Non-T	Chr. band	Start (bp)	Stop (bp)	Strand	Transcript description
**Up-regulated mRNA transcripts**							
FGA	ENSG00000171560	157.6	4q28	154583126	154590766	−	protein-coding
ALB	ENSG00000163631	96.2	4q13	73397114	73421412	+	protein-coding
CRP	ENSG00000132693	84.6	1q23	159712289	159,714,589	−	protein-coding
HP	ENSG00000257017	75.9	16q22	72054566	72,061,056	+	protein-coding
FGB	ENSG00000171564	64.2	4q28	154562956	154572763	+	protein-coding
MMP7	ENSG00000137673	59.2	11q22	102520508	102530753	−	protein-coding
APOA2	ENSG00000158874	54.4	1q23	161222292	161223631	−	protein-coding
ORM1	ENSG00000228278	50	9q31	114323023	114326479	+	protein-coding
APOA1	ENSG00000118137	36.1	11q23	116835751	116837950	−	protein-coding
CLDN1	ENSG00000163347	34.2	3q28	190305701	190322475	−	protein-coding
FOXQ1	ENSG00000164379	31.2	6p25	1312440	1314758	+	protein-coding
TACSTD2	ENSG00000184292	29.2	1p32	58575423	58577773	−	protein-coding
**Up-regulated miRNA transcripts**							
hsa-miR-122	ENSG00000207778	1318.7	18q21	58451068	58451176	+	hsa-miR
hsa-miR-4417	ENSG00000264341	19.9	1p36	5564071	5564143	+	hsa-miR
hsa-miR-1290	ENSG00000221662	16.1	1p34	18897071	18897148	−	hsa-miR
hsa-miR-3687	ENSG00000264063	9.7	21p11	9826203	9826263	+	hsa-miR
hsa-miR-503	ENSG00000208005	9	Xq26	133680358	133680428	−	hsa-miR
hsa-mir-885	ENSG00000216135	8.9	3p25	10394481	10394562	−	hsa-miR
hsa-miR-183	ENSG00000207691	8.6	7q32	129774905	129775014	−	hsa-miR
hsa-miR-224	ENSG00000207621	8.4	Xq28	151958578	151958658	−	hsa-miR
hsa-miR-1246	ENSG00000207584	8.1	2q31	176600980	176601052	−	hsa-miR
**Down-regulated mRNA transcripts**							
CLCA4	ENSG00000016602	−216.3	1p22	87012759	87046437	+	protein-coding
CA1	ENSG00000133742	−142.4	8q21	86239837	86291243	−	protein-coding
MS4A12	ENSG00000071203	−81.7	11q12	60260251	60274903	+	protein-coding
ZG16	ENSG00000174992	−66.5	16p11	29778240	29782973	+	protein-coding
SLC26A3	ENSG00000091138	−65.1	7q31	107405912	107443678	−	protein-coding
CLCA1	ENSG00000016490	−65	1p22	86934051	86965977	+	protein-coding
ACTG2	ENSG00000163017	−61	2p13	73892314	73892314	+	protein-coding
AQP8	ENSG00000103375	−55	16p12	25227052	25240261	+	protein-coding
CA2	ENSG00000104267	−53.6	8q21	85463852	85481493	+	protein-coding
FCGBP	ENSG00000090920	−52	19q13	39863323	39934626	−	protein-coding
GUCA2B	ENSG00000044012	−43.8	1p34	42619092	42621495	+	protein-coding
**Down-regulated miRNA transcripts**							
hsa-miR-133a	ENSG00000207764	−22.5	20q13	61162119	61162220	+	hsa-miR
hsa-miR-133b	ENSG00000199080	−10.8	6p12	52148923	52149041	+	hsa-miR
hsa-miR-10b	ENSG00000207744	−7.5	2q31	176150303	176150412	+	hsa-miR
hsa-miR-215	ENSG00000207590	−7	1q41	220117853	220117962	+	hsa-miR
hsa-miR-363	ENSG00000207572	−5.9	Xq26	134169361	134169473	−	hsa-miR
hsa-miR-143	ENSG00000208035	−5.4	5q32	148808481	148808586	+	hsa-miR
hsa-miR-497	ENSG00000273895	−5.2	17p13	7017911	7018022	−	hsa-miR
hsa-miR-1	ENSG00000174407	−5.2	20q13	62550453	62570764	+	hsa-miR
hsa-miR-138	ENSG00000207954	−4.9	3p21	44114212	44114310	+	hsa-miR
hsa-miR-375	ENSG00000198973	−4.5	2q35	219866362	219866431	−	hsa-miR

q-values < .01; T: tumoral samples; Non-T: non-tumoral samples; hsa-miR: human micro-RNA.

**Table 3:** Top up- and down-regulated mRNAs and miRNAs differentially expressed in colorectal liver metastases (*n* = 19) vs. non-tumoral colorectal tissues (*n* = 10) vs. primary tumors (*n* = 23)

Gene Name	Gene ID	Fold Change T vs. Non−T	Chr. band	Start (bp)	Stop (bp)	Strand	Transcript description
**Up-regulated mRNA transcripts**							
APOA1	ENSG00000118137	10.2	11q23	116835751	116837950	−	protein-coding
HRG	ENSG00000113905	10.0	3q27	186660216	186678240	+	protein-coding
UGT2B4	ENSG00000156096	9.3	4q13	69480165	69526014	−	protein-coding
RBP4	ENSG00000138207	9.0	10q23	93591687	93601744	−	protein-coding
ADH4	ENSG00000198099	8.3	4q22	99123657	99157792	−	protein-coding
SAA2-SAA4	ENSG00000148965	8.1	11p15	18231355	18248674	−	protein-coding
ITIH1	ENSG00000055957	7.9	3p21	52777586	52792068	+	protein-coding
CFHR3	ENSG00000116785	7.6	1q32	196774795	196795406	+	protein-coding
HPX	ENSG00000110169	7.5	11p15	6431038	6442617	−	protein-coding
CFHR2	ENSG00000080910	7.1	1q31	196943759	196959226	+	protein-coding
PLG	ENSG00000122194	7.1	6q26	160702193	160754054	+	protein-coding
**Up-regulated miRNA transcripts**							
hsa-miR-3180-3p	ENSG00000257563	4.8	16p12	18402178	18402271	−	hsa-miR
hsa-miR-3197	ENSG00000263681	4.7	21q22	41167557	41167629	+	hsa-miR
hsa-miR-3178	ENSG00000266232	4.2	16p13	2531922	2532005	−	hsa-miR
hsa-miR-4793	ENSG00000263898	4.1	3p21	48644194	48644280	−	hsa-miR
hsa-miR-4440	ENSG00000266109	4.0	2q37	239068817	239068914	−	hsa-miR
hsa-miR-4486	ENSG00000265210	3.6	11p15	19575310	19575372	+	hsa-miR
hsa-miR-150-star	ENSG00000207782	3.5	19q13	49500762	49500873	−	hsa-miR
hsa-miR-483-5p	ENSG00000207805	3.5	11p15	2134111	2134222	−	hsa-miR
hsa-miR-642b	NA	3.4	19q13	45674932	45675008	−	hsa-miR
hsa-miR-1247	ENSG00000277601	3.4	14q32	101560287	101560422	−	hsa-miR
**Down-regulated mRNA transcripts**							
IGKV1-39	ENSG00000242371	−15.9	2p11	89319,625	89320146	−	protein-coding
IGKC	ENSG00000239975	−15.0	2p12	88857161	89085723	−	protein-coding
IGKV1-27	ENSG00000244575	−13.3	2p11	89213417	89,213,928	−	protein-coding
FABP4	ENSG00000170323	−13.1	8q21	81478419	81483263	−	protein-coding
MYLK	ENSG00000065534	−11.5	3q21	123610049	123884331	−	protein-coding
PLN	ENSG00000198523	−11.3	6q22	118548279	118560730	+	protein-coding
RBPMS2	ENSG00000166831	−11.0	15q22	64739892	64775587	−	protein-coding
CNN1	ENSG00000130176	−9.8	19p13	11538717	11550324	+	protein-coding
SPINK4	ENSG00000122711	−9.8	9p13	33218365	33248567	+	protein-coding
LMOD1	ENSG00000163431	−9.3	1q32	201896452	201946588	−	protein-coding
MYH11	ENSG00000133392	−9.3	16p13	15703135	15857033	−	protein-coding
**Down-regulated miRNA transcripts**							
hsa-miR-363	ENSG00000207572	−5.9	Xq26	134169361	134169473	−	hsa-miR
hsa-miR-1	ENSG00000174407	−5.2	20q13	62550453	62570764	+	hsa-miR
hsa-miR-143	ENSG00000208035	−3.9	5q32	149428918	149429023	+	hsa-miR
hsa-miR-27b	ENSG00000207864	−2.1	9q22	95085435	95085542	+	hsa-miR
hsa-miR-28-5p	ENSG00000207651	−2.0	3q28	188688781	18868866	+	hsa-miR
hsa-miR-99b	ENSG00000207550	−1.7	19q13	51692612	51692681	+	hsa-miR
hsa-miR-152	ENSG00000207947	−1.7	17q21	48037154	48037262	−	hsa-miR
hsa-miR-125b	ENSG00000207971	−1.5	11q24	122099757	122099844	−	hsa-miR
hsa-miR-3064	ENSG00000265695	−1.5	17q23	64500773	64500839	−	hsa-miR
hsa-miR-30d	ENSG00000199153	−1.4	8q24	134804876	134804945	−	hsa-miR

*q-values* < .01; T: tumoral samples; Non-T: non-tumoral samples; hsa-miR: human micro-RNA.

### Functional characterization of deregulated GEP in metastatic sCRC

Analysis of the biological and functional significance of the deregulated GEPs observed in our metastatic colorectal tumors, revealed 58 significantly altered canonical pathways in primary CRC tumors (*vs.* non-tumoral colorectal tissue) and 65 associated with liver mestastases (*vs.* non-tumoral colorectal tissue) (Figure 1). Those pathways specifically altered in primary tumors (Supplementary Table 3) were associated with an abnormally increased expression of genes that are involved in focal adhesion (e.g., PRKCA, CRK, and BCL2), PI3K-Akt signaling (e.g., PHLPP2, PRKCA, PPP2R3A and KIT) and cancer pathways (e.g., COL4A5, LAMC1 and BCL2L1). Conversely, canonical pathways found to be specifically deregulated in liver metastatic samples (Supplementary Table 4) included multiple genes related to intercellular adhesion and the metastatic processes (e.g., IGF1R, PIK3CA, PTEN and EGFR), endocytosis (e.g., the PDGFRA, SMAD2, ERBB3, PML and FGFR2), and the cell cycle (e.g., SMAD2, CCND2, E2F5 and MYC), as well as, the TGFβ signaling pathway (e.g., RHOA, SMAD2, SMAD4, SMAD5, SMAD6, BMPR1A, SMAD7 and MYC), with the FDR values significantly higher for the liver metastases *vs.* primary tumors (≥ 0.0005 to 2.075E-13).

### Validation of tumor markers with high discriminating power between primary tumors and non-tumoral colorectal tissues

The discriminating power of the top mRNAs found to be deregulated (up-regulated and down-regulated) in common in primary sporadic colorectal tumors (*n* = 23) and colorectal liver metastases (*n* = 19) [vs. non-tumoral colorectal tissues (*n* = 10)] was further validated using GEP data from two independent series of sCRC available at the public GEO database (47 primary tumors *vs.* 25 non-tumoral colorectal tissues and 24 liver metastases *vs.* 23 non-tumoral colorectal tissues; Figure 2). In line with those findings described above for our sCRC cases, all primary tumors and non-tumoral samples from the two validation cohorts could be classified as having a sCRC-associated GEP based on overexpression of FOXQ1, MMP7, CLDN1, TACSTD2, COL11A1, KIAA1199, CTHRC1, DUSP27, KRT23, SRPX2 and NFE2L3 and decreased expression of CLCA4, CA1, SLC4A4, AQP8, ZG16, MS4A12, CA2, GUCA2B, CHGA, GUCA2A, CLCA1, SLC26A3 and UGT2B17, respectively. Similarly, 23/24 (96%) liver metastatic samples were also properly classified as distinct from non-tumoral colorectal tissue based on overexpression of APOA1, HRG, UGT2B4, RBP4, ADH4, SAA2-SAA4, ITIH1, CFHR3, HPX, CFHR2 and PLG, while all non-tumoral samples showed decreased expression of IGK1-39, IGKC, IGKV1-27, FABP4, MYLK, PLN, RBPMS2, CNN1, SPINK4, LMOD1 and MYH11 (Figure 2).

### miRNAs genes that potentially regulate gene expression in metastatic sCRC

In order to determine the impact of the miRNAs gene expression signature on the GEP of sCRC tumors, both the miRNA and mRNA gene expression data sets were combined to investigate potential correlations between miRNAs and mRNA genes which are specifically altered in colorectal liver metastases. Evaluation of each pair of potential miRNA-mRNA interacting genes identified potential interactions for 38 inversely correlated and 437 directly correlated (absolute R^2^ value ≥ 0.80; *p* < .0001) pairs of miRNA-mRNA genes. Based on currently available miRNA target prediction algorithms and databases, such interactions corresponded to only three predictable and 4 experimentally validated interactions for the inversely correlated pairs of miRNA-mRNA (Table 4). Of note, both the 4 experimentally validated pairs of mRNA/miRNA genes (miR-20a/DNAJB4, miR-497/PHF19, miR-335/PTPRH and miR-195/SLC23A3) and the 3 predicted miRNA-mRNA pairs (miR-363/LRRC1, miR-3124/PRRC2C and miR-363/ MIER3) were systematically altered in all 19 liver metastatic samples analyzed in this study.

**Table 4:** miRNA-mRNA interactions found in metastatic colorectal cancer by pearson correlation analysis of the expression signal identified for those transcripts differentially and exclusively (vs. primary tumors) expressed in colorectal liver metastases (*n* = 19) as detected by the affymetrix pimeview human gene expression array and the microRNA 3.0 expression array

miRNA	mRNA Gene Name	Gene ID	R^2^	Classification of Interaction	Source of validation/prediction
hsa-miR-20a	DNAJB4	ENSG00000162616	−0.84	Validated	miRTarBase
hsa-miR-497	PHF19	ENSG00000119403	−0.83	Validated	miRTarBase
hsa-miR-335	PTPRH	ENSG00000080031	−0.82	Validated	miRTarBase, DIANAmT
hsa-miR-195	SLC23A3	ENSG00000213901	−0.82	Validated	miRTarBase
hsa-miR-363	LRRC1	ENSG00000137269	−0.89	Predicted	MiRanda
hsa-miR-3124	PRRC2C	ENSG00000117523	−0.86	Predicted	Targetscan
hsa-miR-363	MIER3	ENSG00000155545	−0.81	Predicted	Targetscan
hsa-miR-10b-star	NBPF1	ENSG00000219481	−0.85	Not known	
hsa-miR-30a	KIF4A	ENSG00000090889	−0.85	Not known	
hsa-miR-31	QPRT	ENSG00000103485	−0.85	Not known	
hsa-miR-1910	RASL12	ENSG00000103710	−0.84	Not known	
hsa-miR-30a	SKA1	ENSG00000154839	−0.84	Not known	
hsa-miR-195	SKA1	ENSG00000154839	−0.83	Not known	
hsa-miR-3162	TFCP2L1	ENSG00000115112	−0.83	Not known	
hsa-miR-486	SPC24	ENSG00000161888	−0.83	Not known	
hsa-miR-497	SKA1	ENSG00000154839	−0.83	Not known	
hsa-miR-885	RNF43	ENSG00000108375	−0.83	Not known	
hsa-miR-195	F2RL1	ENSG00000164251	−0.82	Not known	
hsa-miR-195	KIF4A	ENSG00000090889	−0.82	Not known	
hsa-miR-30a	BORA	ENSG00000136122	−0.82	Not known	
hsa-miR-4484	CASP1	ENSG00000137752	−0.82	Not known	
hsa-miR-497	HMMR	ENSG00000072571	−0.82	Not known	
hsa-miR-497	KIF4A	ENSG00000090889	−0.82	Not known	
hsa-miR-497	PTTG1	ENSG00000164611	−0.82	Not known	
hsa-miR-195	CKMT1A	ENSG00000223572	−0.81	Not known	
hsa-miR-20a	PCSK5	ENSG00000099139	−0.81	Not known	
hsa-miR-223	PDE9A	ENSG00000160191	−0.81	Not known	
hsa-miR-363	MRPS17	ENSG00000239789	−0.81	Not known	
hsa-miR-4443	RRP1	ENSG00000160214	−0.81	Not known	
hsa-miR-150	TFCP2L1	ENSG00000115112	−0.80	Not known	
hsa-miR-20a	RNF152	ENSG00000176641	−0.80	Not known	
hsa-miR-20a	SOCS2	ENSG00000120833	−0.80	Not known	
hsa-miR-30a	MS4A8	ENSG00000166959	−0.80	Not known	
hsa-miR-363	CNBP	ENSG00000169714	−0.80	Not known	
hsa-miR-363	UQCRC2	ENSG00000140740	−0.80	Not known	
hsa-miR-497	CKMT1A	ENSG00000223572	−0.80	Not known	
hsa-miR-497	F2RL1	ENSG00000164251	−0.80	Not known	
hsa-miR-497	HDHD2	ENSG00000167220	−0.80	Not known	
hsa.miR.378g	PMEPA1	ENSG00000124225	−0.80	Not known	

*p-values <.0001; R*^2^*: Pearson correlation coefficient.*

## DISCUSSION

In the present study we investigated the expression of both coding mRNA and non-coding RNA genes -including miRNA, small nucleolar and large intergenic RNAs- in paired primary tumor and liver metastases samples from primary colorectal carcinoma patients. To the best of our knowledge, this is the first study in which the overall coding and non-coding GEP of sCRC liver metastases are compared with their paired primary tumors and non-tumoral colorectal tissues, by high-resolution arrays. Overall, primary tumors and their paired metastases frequently revealed many genes to be altered in common at both sites; these findings support the existence of a very close genomic relationship between the GEP of primary colorectal tumors and their paired liver metastases, as it has also been suggested to occur at the DNA level [16]. Deregulated expression observed in common in both groups of (paired) tumor samples included increased expression of genes linked to liver metastatic sCRC, tumor development, growth (TACSTD2) [17] and invasiveness (FOXQ1, MMP7 and CLDN1) [18, 19], together with decreased expression of genes related to differentiation (CLCA4, CLCA1 and AQP8) [20, 21], apoptosis (CA1) [22], immunity (ZG16) [23], and protection (FCGBP and CA2) [24] of CRC cells. Thus, the functional networks most strongly affected in primary tumors included those involved in focal adhesion, PI3K-Akt and ErbB signaling and other cancer pathways, FcδR-mediated phagocytosis, endocytosis, bacterial invasion of epithelial cells, plus the TP53 signaling pathway, all of which have been previously shown to be directly involved in the development and progression of sCRC [25–29].

Another functional gene network which was also frequently altered in sCRC liver metastases was the TGFβ signaling pathway, a network that plays a critical role in the regulation of cell growth, differentiation and development in a broad range of neoplasms [7]. In fact, such findings have led to the speculation that TGFβ signaling might be responsible, at least in part, for the aggressiveness of CRC tumors in our series, through an increased invasive capacity which is a prerequisite for the settlement and growth of distant metastasis [18]. In this regard, recent studies in which the miRNAS signature of metastatic CRC has been investigated, have also identified a network of regulatory miRNA-driven interactions that regulate expression of key genes associated with TGFβ signaling and metastasis, including the miR-378 gene family [30]. In line with these observations, here we found the miRNA-378c, −378d, −378f, −378i, −378g and −378e to be among the most strongly down-regulated miRNAs in primary CRC tumors. Zhang *et al.* [30], evaluated the levels of miR-378 in CRC cell lines and 84 paired CRC cancer and normal adjacent mucosa tissues, their results highlighting miR-378 expression to be an independent prognostic factor potentially due to its ability to inhibit cell growth and invasion in CRC. Most interestingly, here we found that miR-378g could directly target PMEPA1, a gene that plays a relevant role in the TGF-β signaling pathway [31, 32]. In this regard, it has been recently shown that the TGF-β signaling regulator PMEPA1 suppresses prostate cancer metastases to bone [32]. Despite this, the impact of an altered PMEPA1 expression on CRC, as well as the potential underlying mechanisms for such association, still remain to be investigated. Moreover, several miRNA whose expression was strongly down-regulated in metastatic CRC samples, are also involved in the TGFβ signaling pathway; these included the miR-133a, miR-133b, miR-1 and miR-375 miRNAs [33].

Previous studies in which the miRNA signature of metastatic CRC has been investigated, have identified a unique pattern of miRNA which are differentially expressed in metastatic tumors and that include a network of regulatory miRNA-driven interactions involved in the expression of key genes associated with the epithelial to mesenchymal transition (EMT) and tumor metastasis, such as the miR-200 gene family [15]. However, in these studies the transcriptional profiles of the metastatic liver was defined though comparison of the gene expression profile of CRC metastatic tissues against normal liver, despite the background GEP of the tumor present in the liver derives from cells of the (tumoral) intestinal mucosa and not from liver tissues. Similarly, Lin *et al.* [34] using supervised SAM analysis, identified 963 unique genes to be significantly overexpressed in colorectal liver metastases vs. primary CRC (and normal liver tissue). However, several of the differently expressed genes are known to be up-regulated in normal liver vs colon tissues (i.e.: ARG1, BAAT, BHMT, CDO1, CRHBP, F11, F5, FGL1, HAMP, HAMP and ITIH2).

Of note, miR-122 emerged as the most up-regulated miRNA gene in liver metastases vs. both non-tumoral colorectal mucosa and primary CRC tissues. Overexpression of miR-122 and the simultaneus suppression of its target gene, *cationic amino acid transporter 1*, has been shown to be involved in the development of colorectal liver metastasis [35]. However, Pizzini et al. [36] have previously suggest that overexpression of miR-122 in CRC liver metastatic tissues could be due to the presence of normal residual liver tissue. Based on an extensive analysis of other liver associated genes, our data suggests this might not be the case and that miR-122 could in fact the overexpression by metastatic liver cells. In this regard, it should be noted that an interesting cross-talk has been reported between hepatocarcinoma cells, through which cells expressing miR-122 send this miRNA via microvesicles to inhibit the proliferation of miR-122 deficient cells. In a reciprocal process, miR-122 deficient cells secrete insulin-like growth factor to decrease miR-122 expression in miR-122 expressing cells [37].

Our results also showed miR-10b to be down-regulated in liver metastatic tissues vs non-tumoral colorectal mucosa. The expression levels of this miRNA in metastataic samples (vs. primary tumors) have previously been correlated with CRC patients survival [36]. In this regard, Hur et al have recently identified a metastasis-specific microRNA signature that includes high miR-10b expression in primary tumors, to be an independent predictor of distant metastasis [38]. In addition, it is also well known that miR-10b functionally contributes to tumor invasion and metastasis in breast cancer [39], and to a worse prognosis in both esophageal [40] and pancreatic cancer patients [41]. Despite all the above, delineation of the specific functional effects of miRNA levels on the expression of mRNA transcripts still remains a challenge. Here we identified several miRNAs whose expression levels were significantly correlated with the amount of mRNA of specific genes. Among other pairs of miRNA-mRNA genes identified, the miR-20a and the DNAJB4 gene transcript emerged in our series, as significantly correlated. DNAJB4 (DnaJ heat shock protein family member B4) is a chaperone with a strong tumor suppressor effect in CRC whose down-regulation has been associated with patient outcome [42]. In this regard, miR-20a has also been shown to induce EMT, to regulate the migration and invasion of SW480 cells [43] and to contribute to an increased chemoresistance of CRC [44]. Of note, here we did not find any (inverse) correlation between the levels of expression of miR-20a and the mRNA levels of other genes reported to be altered IN parallel in CRC such as the PCSK5, RNF152 and SOCS2 genes [45–47]. Other miRNAs which have been found to be altered in CRC include the miR-497, which might mediate overexpression of genes involved in cell proliferation (e.g. PHF19, SKA1, KIF4A) and genes associated with cell migration and invasion (e.g. PTTG1, F2RL1) as well as genes related to the transfer of high energy phosphate from mitochondria, potentially related to the failure to eliminate cancer cells via apoptosis (e.g. CKMT1A) [48, 49].

Interestingly, clearcut discrimination between primary CRC, liver metastases and non-tumoral colorectal tissue could be obtained via those mRNAs found to be most strongly up- and down-regulated exclusively in primary tumors and their liver metastases, as also confirmed in two external series of 47 metastatic CRC [50] and 24 colorectal liver metastases [36]. These results indicate that these gene signatures could potentially serve in the future as prior knowledge for the discovery of biomarker candidates for primary sCRC tumors for early diagnosis, more efficient treatment and/or monitoring of metastatic CRC. In line with this hypothesis, strong expression of the MMP7, CLDN1, TACSTD2, CTHRC1, KRT23 and SRPX2 genes has been previously reported at the protein level in CRC tissues, their expression been also associated with tumor progression and an increased angiogenesis [51]; of note most of these proteins (i.e.: MMP7, TACSTD2, CTHRC1 and KRT23) have also been found to be secreted and to be present in both tumor tissues and the plasma from CRC patients [52]. Therefore, secretion of these proteins outside the tumor cell supports the potential utility of these genes as serum biomarkers for early diagnosis and monitoring of CRC patients. However, additional studies are still required to further validate in non-metastatic CRC the mRNA and miRNA expression profiles here described.

In summary, here we report on a high number of a mRNA and miRNA that are altered in common in (paired) primary and metastatic colorectal tumors, and that allow clearcut distinction between the tumoral and normal colon tissues based on a few mRNAs potentially critical in either the metastatic process and/or the adaption of metastatic cells to the metastatic niche/environment. In this regard, activation of genes associated with the TGFβ signaling pathway emerged as potentially relevant candidate genes in CRC metastasis.

## MATERIALS AND METHODS

### Patients and samples

Tissue specimens from 23 sporadic colorectal adenocarcinomas and 19 paired liver metastases (*n* = 42 samples) were obtained from 23 patients with metastatic lesions susceptible of being resected (16 males and 7 females; median age of 66 years, ranging from 48 to 80 years) after informed consent had been given by each subject; in four patients no metastatic liver tissue was available. The studied cases corresponded to 23 consecutive metastatic colorectal cancer patients. Patients underwent surgical resection of both primary and metastatic tumor tissues at the Department of Surgery of the University Hospital of Salamanca (Salamanca, Spain), prior to any cytotoxic therapy was given. Tumor diagnosis and classification was performed according to the AJCC criteria [53]. According to tumor grade, 11 cases were classified as well-differentiated tumors, 10 as moderately- and 2 as poorly-differentiated carcinomas. In all cases, histopathological grade was systematically confirmed in a second independent evaluation by an experienced pathologist (M.A.A and OB). Median follow-up at the moment of closing the study was of 25 months (range: 11–39 months).

Primary tumors were localized in the rectum (*n* = 11) and either in the right (cecum, ascending or transverse) or the left (descending and sigmoid) colon (*n* = 12). The mean size of the primary tumors was of 5.4 ± 2.0 cm with the following distribution according to their TNM stage at diagnosis [54]: T3N0M0, 2 cases; T3N1M1, 8; T3N1M0, 1; T3N0M1, 3; T3N2M1, 2; T4N0M1, 2; T4N1M1, 1; T4N2M1, 2; T2N0M0, 1 and; T3N2M0, 1 case. Eighteen liver metastases were synchronous and 5 were metachronous metastases. The mean size of liver metastases was of 3.6 ± 2.1 cm. The most relevant clinical and laboratory data for each individual metastatic colorectal cancer patient studied is summarized in Supplementary Table 1.

Colorectal tissue samples not required for diagnostic purpose were collected immediately after surgical resection, snap frozen and stored in OCT at −80°C (Tumor Biobank of the University Hospital of Salamanca, Red de Bancos de Tumores de Castilla y León, Salamanca, Spain). Once the histopathological diagnosis had been established, sections from paraffin-embedded tissue samples were cut from three different areas representative of the tumor tissue with > 70% tumor cell infiltration by hematoxylin-eosin staining, after excluding stroma-enriched tumor areas. In order to enrich the tumor cells, the neighbour areas of those containing ≥ 70% tumor cells, as well as liver tissue samples were then microdissected from the frozen tumor tissue samples stored in OCT, following the pathologist criteria. Normal mucosa samples (*n* = 9) were taken at a minimum distance of 10 cm from the tumor site. The study was approved by the local ethics committee (University Hospital of Salamanca, Salamanca, Spain).

### RNA extraction and gene expression profiling (GEP) microarray studies

For GEP, sample preparation was performed as described in the Affymetrix GeneChip Expression Analysis Manual (Santa Clara, CA, USA). Briefly, each frozen tissue (≥ 0.3 g) was crushed to powder at cryogenic temperatures and homogeneized in Trizol (Life Technologies Inc, Rockville, MD). Total RNA was then extracted using the miRNeasy mini kit according to the instructions of the manufacturer (Qiagen, Valencia, CA); subsequently, the quality and integrity of the RNA was evaluated in an Agilent 2100 Bioanalyzer (Agilent Technologies Inc, Santa Clara, CA). Total RNA (100–1000 ng) from both tumoral and non-tumoral colorectal tissues was hybridized to both the Affymetrix PimeView Human Gene Expression and the microRNA 3.0 Expression arrays, following the instructions of the manufacturer. Fluorescence signals were detected using the GeneChip Scanner 3000 7G (Affymetrix) and data stored as .CEL files. GEP raw data has been deposited in the Gene Expression Omnibus (GEO) database with the GSE81582 accession number, includes data based on sets of 49,395 and 5,683 probes for the Affymetrix PimeView Human Gene Expression microarray and the microRNA 3.0 microarray, respectively.

For data analysis, GEP raw data was normalized with the Robust Multi-array Average (RMA) algorithm, which included sequential background correction, intra- and inter-microarray well normalization, probe set summarization and calculation of expression signals [55]. Unsupervised classification of samples and genes was performed by MultiDimensional Scaling (MDS) and hierarchical clustering analyses (HCA) based on the expression signal detected for each gene for each probe set, and the Simfit statistical software (http://www.simfit.org.uk/). Clustering was run using Euclidean distances and the linkage method group average. Differentially expressed (miRNA and mRNA) genes between tumoral and non-tumoral samples were identified by supervised two-class unpaired Significance Analysis of Microarray (SAM) [56] based on a *q*-value cut off ≤ .01 and an absolute fold change cutoff ≥ 2.0. Differentially expressed mRNA and miRNA in pairwise group comparisons, involving groups of samples paired per patient (*n* = 19 paired liver metastasis vs 19 primary tumor samples) were calculated with SAM using a two-class pair wise design. The false discovery rate (FDR) cutoff for statistical significance was set at values < .01.

For the identification of miRNA candidates acting as gene-regulators in colorectal samples, Pearson correlation analysis was performed to identify significant associations between deregulated miRNA and mRNA gene transcripts in both primary colorectal tumors (*vs*. non-tumoral colorectal tissues) and colorectal liver metastases (*vs*. non-tumoral colorectal tissues) through the psych R-package, based on an adjusted FDR of ≤ .05. Each potential miRNA-mRNA interaction identified was subsequently evaluated against databases of experimentally validated miRNA interactions (TarBase 6.0 and miRWalk-database) and miRNA target prediction tools (DIANA-microT-CDS v5.0, miRWalk-database and miRecords) [57, 58]. Functional enrichment analysis of deregulated genes, as well as analysis of canonical pathways, was based on the use of the WebGestalt suite (http://bioinfo.vanderbilt.edu/webgestalt).

### Enrichment of kyoto encyclopedia of genes and genomes (KEGG) pathways

To identify significantly altered KEGG pathways for each comparison performed, we applied the hypergeometric distribution statistical test was applied to calculate the probability of overlap of the deregulated transcripts, targets of significantly differentially expressed miRNAs, and gene expression involved in KEGG pathways; *p*-values were corrected using the Benjamini-Hochberg.

### Validation of miRNA expression profiles by quantitative real-time PCR (QR-PCR)

TaqMan miRNA expression assays were used to validate GEP in the same samples that were used for the microarray studies, via the Step One Plus Real-Time PCR System -Applied Biosystems (ABI), Foster City, CA, USA- according to the manufacturer's instructions. The assays ID for the miRNAs studied were as follows: 002245 (hsa-miR-122), 2246 (hsa-miR-133a), 002249 (hsa-miR-143) and 461910 (hsa-miR-4417). Each PCR was carried out in duplicate in a final volume of 10 uL using the TaqMan Fast Universal Mastermix (ABI) and the following cycling parameters: incubation at 95°C (20s), followed by 50 cycles at 95°C (1s) and an incubation at 60°C (20s). miRNA expression data was normalized against the RNU43 internal control, and it was further analyzed using the StepOne software (v2.0; ABI). The relative amounts of the quantified miRNAs were calculated using the following equation: 2^−ΔCT^ (ΔC_T_ = C_T_ GENE-C_T_ RNU43) expressed as arbitrary units (AU). For all genes evaluated, RQ-PCR results showed a high degree of correlation with microarray data (r^2^ ≥ 0.79, *p* < .0001; Supplementary Figure 2).

### External validation series of sCRC tumors

External validation of the predictive value of those differentially expressed genes found in our series to discriminate between primary tumors and non-tumoral colorectal tissues, was performed in a group of previously reported metastatic sCRC patients (*n* = 47) from whom GEP array data files (Affymetrix Human Genome U133 Plus 2.0 Array) are publicly available at the GEO database (accession number GSE21510) [50]. Additionally, to discriminate between liver metastases and non-tumoral colorectal tissues, we performed an external validation in another group of previously reported metastatic sCRC patients (*n* = 24) from whom GEP array data files (Affymetrix Human Exon 1.0 ST Array) are also publicly available at the GEO database (accession number GSE35834) [36].

Downloaded data CEL files were normalized using the RMA algorithm and overlapping probe sets were defined on the basis of probe specificity, using the GATExplorer server [59]. Probe sets with the best specificity to the interrogated genes were selected, and the expression signals detected for each gene for each probe set were further analyzed using the column metric preserving biplot assay [60] implemented in the SIMFIT statistical software (http://www.simfit.org.uk/).

### External validation series of DEFB1, COL12A1 and PTGER3 gene expression

External validation of those genes found to be differentially expressed in our series between primary tumors and their corresponding liver metastases (DEFB1, COL12A1 and PTGER3), was performed in a group of previously reported metastatic sCRC patients (*n* = 18) from whom RNA-seq data files (Illumina, CA) are publicly available at the GEO database (accession number GSE50760) [61]; in line with our observations, these results confirmed the existence of significant differences in the expression of the DEFB1, COL12A1 and PTGER3 genes between paired primary tumor and their corresponding liver metastases (Supplementary Figure 3).

### Other statistical methods

The Mann-Whitney *U* test and a linear regression model were used to evaluate the statistical significance of differences observed between groups and to explore the degree of correlation between different variables, respectively (SPSS version 15.0; IBM; NY; USA). *P-values* ≤ .05 were considered to be associated with statistical significance.

## SUPPLEMENTARY MATERIALS FIGURES AND TABLES








