# Supplementary material for: Genomic characterization of liver metastases from colorectal cancer patients
Source: Oncotarget. 2016 Sep 20;7(45):72908–22. doi: 10.18632/oncotarget.12140 (PMC5341953; doi:10.18632/oncotarget.12140)
Supplement: Supplementary file 1 [file oncotarget-07-72908-s001.pdf]

Genomic characterization of liver metastases from colorectal cancer patients

Supplementary Materials

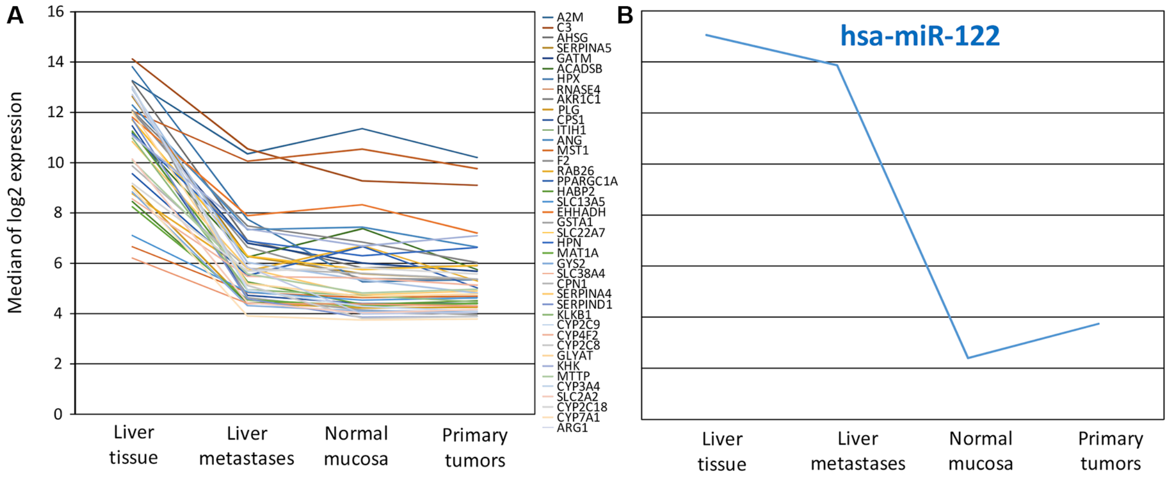

Supplementary Figure S1: Levels of expression of 41 liver-associated coding and non-coding genes (selected from the Tissue-specific Gene Expression and Regulation database) (Panel A) and miR-122 (panel B) in normal liver tissue (*n* = 5), CRC liver metastases (*n* = 19), normal colorectal mucosa (*n* = 9) and primary CRC tumors (*n* = 23), as identified through analysis of the GEP of coding and non-coding RNAs.

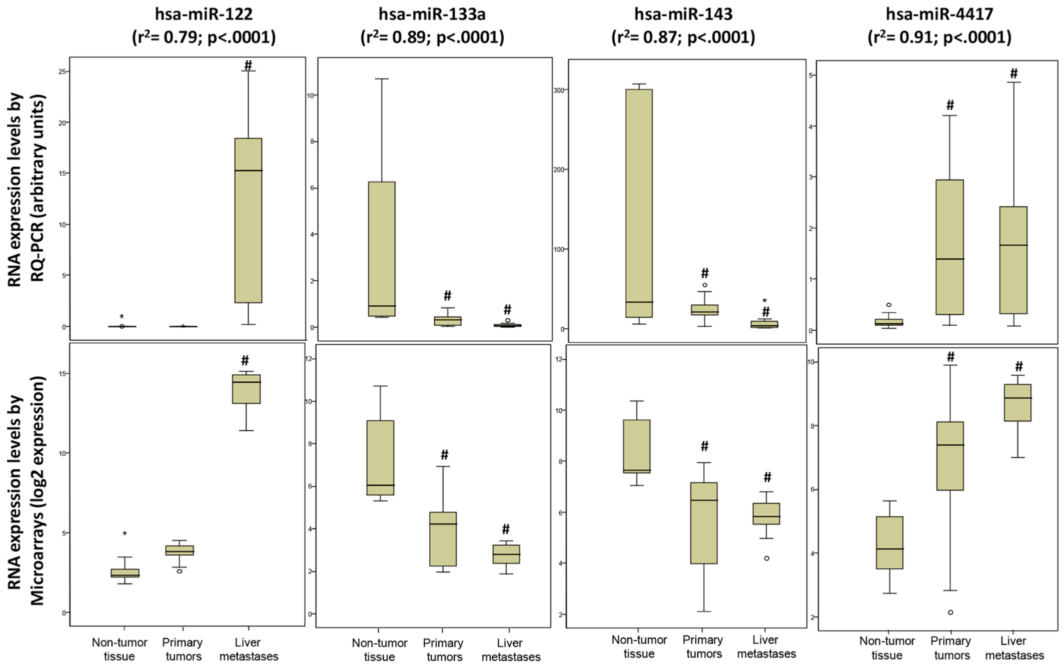

Supplementary Figure S2: Expression levels of the hsa-mir-122, hsa-mir-133a, hsa-mir-143 and hsa-mir-4417 miRNA transcripts in normal colorectal mucosa (*n* = 9), primary CRC tumors (*n* = 23) and CRC liver metastases (*n* = 19) as assessed by the RQ-PCR method used to validate GEP microarray data. Notched boxes represent 25th and 75th percentile values. The line in the middle and vertical lines correspond to the median values and 95% confidence intervals, respectively. Outliers (values that are between 1.5 and 3 times the interquartile range) are marked with a circle and extreme cases (values that are more than three times the interquartile range) with an asterisk. Values of the correlation analysis performed between the RQ-PCR and the microarrays expression levels of the CRC-deregulated are showed in parenthesis. "#" is shown when the expression levels of miRNAs analyzed in the primary tumors and liver metastases are significantly different from the normal mucosa ($p < .001$).

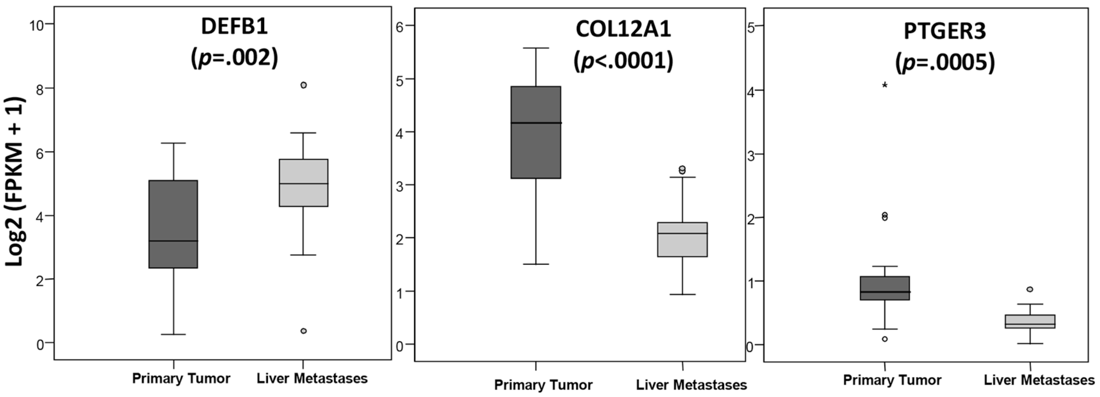

Supplementary Figure S3: Comparative analysis of the DEFB1, COL12A1 and PTGER3 gene expression by the RNA-seq method between 18 paired primary tumors and their synchronous liver metastases from an independent external validation dataset (GEO database, accession number GSE50760). Notched boxes represent 25th and 75th percentile values. The line in the middle and vertical lines correspond to the median values and 95% confidence intervals, respectively. Outliers (values that are between 1.5 and 3 times the interquartile range) are marked with a circle and extreme cases (values that are more than three times the interquartile range) with an asterisk.

Supplementary Table S1: Detail clinical and biological characteristics of each individual metastatic colorectal cancer patient analyzed (*n* = 23)

Cases ID	Gender	Age (year)	CEA serum levels (ng/ml)	Site of primary tumor	Histological grade	Primary tumor size (cm)	TNM stage	N. of metastasis	*KRAS* mutational status	MSI mutational status*
1	M	69	7.6	Left colon	Well	3.0	T3N0M1	1	WT	Normal
2	M	57	30.9	Left colon	Well	6.0	T3N1M1	> 3	WT	Normal
3	M	77	244.9	Left colon	Well	5.5	T3N1M1	1	WT	Normal
4	F	80	85.3	Left colon	Well	3.0	T4N0M1	1	WT	Normal
5	M	64	256.0	Left colon	Well	7.0	T3N0M1	> 3	WT	Normal
6	M	75	589.2	Rectum	Well	4.0	T3N1M1	2	WT	Normal
7	F	62	139.0	Rectum	Well	7.5	T3N0M1	1	WT	Normal
8	M	63	23.2	Rectum	Well	7.0	T3N2M1	1	WT	Normal
9	M	77	58.3	Rectum	Well	9.0	T3N1M1	1	WT	Normal
10	M	64	5.4	Rectum	Well	7.0	T3N0M0	1	WT	Normal
11	M	66	3.7	Rectum	Well	8.5	T3N0M0	1	WT	Normal
12	M	76	43.9	Right colon	Moderate	5.5	T3N1M1	> 3	WT	Normal
13	M	62	155.2	Right colon	Moderate	3.0	T3N2M1	3	G12A	Normal
14	F	58	501.0	Left colon	Moderate	5.0	T4N2M1	> 3	WT	Normal
15	F	75	1145.0	Left colon	Moderate	9.0	T4N1M1	> 3	WT	Normal
16	M	61	1.2	Left colon	Moderate	3.0	T2N0M0	1	WT	Normal
17	F	76	149.8	Rectum	Moderate	2.5	T3N1M1	3	WT	Normal
18	F	49	6.8	Rectum	Moderate	6.5	T3N1M1	2	NT	Normal
19	M	74	110.0	Rectum	Moderate	5.0	T4N0M1	> 3	WT	Normal
20	M	73	6.4	Rectum	Moderate	5.5	T3N1M0	1	WT	Normal
21	M	61	2.3	Rectum	Moderate	5.0	T3N2M0	1	G12A	Normal
22	F	48	32.9	Left colon	Poor	4.0	T4N2M1	> 3	WT	Normal
23	M	72	45.4	Left colon	Poor	4.0	T3N1M1	2	WT	Normal

M: male; F: female; CEA: carcinoembryonic antigen; WT: wild-type; NT: NT: not tested.
*MLH1, MSH2 and MSH6 mismatch repair proteins in the nucleus and adjacent non-neoplastic tissue elements were analyzed by immunohistochemistry.

Supplementary Table S2: mRNAs and miRNAs up- and down-regulated in colorectal liver metastases (*n* = 19) vs. primary tumors (*n* = 19), according to paired test (FDR < .01). See Supplementary_Table_S2

Supplementary Table S3: Most representative canonical pathways and genes involved in primary sporadic tumors (*n* = 23) as identified through analysis of the GEP of coding and non-coding RNAs (FDR < .01). See Supplementary_Table_S3

Supplementary Table S4: Most representative canonical pathways and their corresponding genes involved in colorectal liver metastases (*n* = 19) as identified through analysis of the GEP of coding and non-coding RNAs (FDR < .01). See Supplementary_Table_S4
