# Supplementary material for: Genomic characterization of liver metastases from colorectal cancer patients
Source: Oncotarget. 2016 Sep 20;7(45):72908–22. doi: 10.18632/oncotarget.12140 (PMC5341953; doi:10.18632/oncotarget.12140)
Supplement: Supplementary file 2 [file oncotarget-07-72908-s002.docx]

| **Gene Name** | **Gene ID** | **Fold change**  **in LM vs. PT** | **Chr. band** | **Start (bp)** | **Stop (bp)** | **Strand** | **Transcript**  **description** |
| --- | --- | --- | --- | --- | --- | --- | --- |
| ***Up-regulated mRNA transcripts*** | |  |  |  |  |  |  |
| FGA | ENSG00000171560 | 129.8 | 4q28 | 154583126 | 154590766 | - | protein-coding |
| ALB | ENSG00000163631 | 85.5 | 4q13 | 73397114 | 73421412 | + | protein-coding |
| CRP | ENSG00000132693 | 80.8 | 1q23 | 159712289 | 159,714,589 | - | protein-coding |
| HP | ENSG00000257017 | 51.5 | 16q22 | 72054566 | 72,061,056 | + | protein-coding |
| FGB | ENSG00000171564 | 48.6 | 4q28 | 154562956 | 154572763 | + | protein-coding |
| APOA2 | ENSG00000158874 | 41.1 | 1q23 | 161222292 | 161223631 | - | protein-coding |
| APOA1 | ENSG00000118137 | 34.7 | 11q23 | 116835751 | 116837950 | - | protein-coding |
| APCS | ENSG00000132703 | 32.4 | 1q21 | 159587825 | 159588871 | + | protein-coding |
| SERPINA3 | ENSG00000196136 | 28.9 | 14q32 | 94612377 | 190322475 | + | protein-coding |
| ITIH2 | ENSG00000151655 | 28.1 | 10p15 | 7703269 | 7749520 | + | protein-coding |
| FGG | ENSG00000171557 | 25.5 | 4q28 | 154604134 | 154612967 | - | protein-coding |
| APOB | ENSG00000084674 | 25.2 | 2p24 | 20999218 | 21044073 | - | protein-coding |
| SERPINA1 | ENSG00000197249 | 24.2 | 14q32 | 94376747 | 94390693 | - | protein-coding |
| IGFBP1 | ENSG00000146678 | 22.7 | 7p12 | 45888357 | 45893668 | + | protein-coding |
| ORM1 | ENSG00000228278 | 21.9 | 9q31 | 114323023 | 114326479 | + | protein-coding |
| TF | ENSG00000091513 | 17.5 | 3q22 | 133661998 | 133779006 | + | protein-coding |
| CP | ENSG00000047457 | 16.1 | 3q23 | 149162410 | 149222055 | - | protein-coding |
| APOC3 | ENSG00000110245 | 14.5 | 11q23 | 116829706 | 116833072 | + | protein-coding |
| FGL1 | ENSG00000104760 | 14.3 | 8p22 | 17864380 | 17910365 | - | protein-coding |
| ORM2 | ENSG00000228278 | 14.2 | 9q32 | 114329789 | 114333256 | + | protein-coding |
| ITH4 | ENSG00000055955 | 11.1 | 3p21 | 52812975 | 52831479 | - | protein-coding |
| TM4SF4 | ENSG00000169903 | 10.8 | 3q25 | 149473974 | 149503394 | + | protein-coding |
| ITIH3 | ENSG00000162267 | 9.8 | 3p21 | 52794768 | 52809009 | + | protein-coding |
| FMO3 | ENSG00000007933 | 7.8 | 1q24 | 171090877 | 171117821 | + | protein-coding |
| CYP2C9 | ENSG00000138109 | 7.7 | 10q23 | 94938658 | 94989391 | + | protein-coding |
| VNN1 | ENSG00000112299 | 7.2 | 6q23 | 132680858 | 132714055 | - | protein-coding |
| F5 | ENSG00000198734 | 5.2 | 1q23 | 169511954 | 169586588 | - | protein-coding |
| COLEC11 | ENSG00000118004 | 4.3 | 2p25 | 3594832 | 3644644 | + | protein-coding |
| FXYD2 | ENSG00000137731 | 1.9 | 11q23 | 117800844 | 117828698 | - | protein-coding |
| TNFRSF10D | ENSG00000173530 | 1.7 | 8p21 | 23135588 | 23164030 | - | protein-coding |
| ABCC3 | ENSG00000108846 | 1.4 | 17q22 | 50634777 | 50692252 | + | protein-coding |
| CTAGE5 | ENSG00000150527 | 1.4 | 14q13 | 39265272 | 39388513 | + | protein-coding |
| ACAD10 | ENSG00000111271 | 1.4 | 12q24 | 111686053 | 111757107 | + | protein-coding |
| PPP1R35 | ENSG00000160813 | 1.4 | 7q22 | 100435277 | 100436615 | - | protein-coding |
| MAP3K9 | ENSG00000006432 | 1.3 | 14q24 | 70722526 | 70809534 | - | protein-coding |
| KIAA0319L | ENSG00000142687 | 1.3 | 1p34 | 35433490 | 35557950 | - | protein-coding |
| LPCAT3 | ENSG00000111684 | 1.3 | 12p13 | 6976185 | 7018538 | - | protein-coding |
| RABGGTA | ENSG00000100949 | 1.2 | 14q11 | 24265538 | 24271739 | - | protein-coding |
| ***Up-regulated miRNA transcripts*** | | |  |  |  |  |  |
| hsa-miR-122 | ENSG00000207778 | 669.9 | 18q21 | 58451068 | 58451176 | + | hsa-miR |
| hsa-miR-4322 | ENSG00000264341 | 2.2 | 19p13.2 | 10230413 | 10230485 | + | hsa-miR |
| ***Down-regulated mRNA transcripts*** | |  |  |  |  |  |  |
| ACTG2 | ENSG00000163017 | -11.7 | 2p13 | 73892314 | 73892314 | + | protein-coding |
| PCDH18 | ENSG00000189184 | -4.4 | 4q31 | 137518918 | 137532498 | - | protein-coding |
| DIO2 | ENSG00000211448 | -3.6 | 14q31 | 80197525 | 80387757 | - | protein-coding |
| RGMA | ENSG00000182175 | -3.3 | 11q12 | 60260251 | 60274903 | + | protein-coding |
| FOXF1 | ENSG00000103241 | -2.8 | 16q24 | 86510527 | 86515418 | + | protein-coding |
| PDZD2 | ENSG00000133401 | -2.7 | 5p13 | 31639363 | 32110932 | + | protein-coding |
| NKX2-3 | ENSG00000119919 | -2.5 | 10q24 | 99532933 | 99536524 | + | protein-coding |
| FBN2 | ENSG00000138829 | -2.3 | 5q23 | 128257909 | 128659185 | - | protein-coding |
| FZD8 | ENSG00000177283 | -1.9 | 10p11 | 35638249 | 35642278 | - | protein-coding |
| PBX1 | ENSG00000185630 | -1.9 | 1q23 | 164555584 | 164899296 | + | protein-coding |
| OXCT1 | ENSG00000083720 | -1.9 | 5p13 | 41730065 | 41870689 | - | protein-coding |
| SGCB | ENSG00000163069 | -1.7 | 4q12 | 52020695 | 52038482 | - | protein-coding |
| RHOA | ENSG00000067560 | -1.6 | 3p21 | 49359136 | 49412998 | - | protein-coding |
| C12orf4 | ENSG00000047621 | -1.3 | 12p13 | 4487728 | 4538508 | - | protein-coding |
|  |  |  |  |  |  |  |  |

**Supplementary Table 2**. mRNAs and miRNAs up- and down-regulated in colorectal liver metastases (n=19) *vs.* primary tumors (n=19), according to paired test (FDR<.01).

PT: primary tumors; LM: liver metastases; hsa-miR: human micro-RNA.
