# Supplementary material for: Genomic characterization of liver metastases from colorectal cancer patients
Source: Oncotarget. 2016 Sep 20;7(45):72908–22. doi: 10.18632/oncotarget.12140 (PMC5341953; doi:10.18632/oncotarget.12140)
Supplement: Supplementary file 3 [file oncotarget-07-72908-s003.docx]

**SUPPLEMENTARY TABLE 3.** Most representative canonical pathways and genes involved in primary sporadic tumors (n=23) as identified through analysis of the GEP of coding and non-coding RNAs (FDR < .01).

| **Canonical Pathways** | **miRNAs ID** | **Gene ID** | | **FDR** |  |
| --- | --- | --- | --- | --- | --- |
| Focal adhesion | hsa-miR-215, hsa-miR-503, hsa-miR-139-5p, hsa-miR-133a, hsa-miR-378c, hsa-miR-378d, hsa-miR-422a, hsa-miR-375, hsa-miR-378f, hsa-miR-378i, hsa-miR-1290, hsa-miR-378g, hsa-miR-133b, hsa-miR-592, hsa-miR-378e, hsa-miR-1246, hsa-miR-486-5p, hsa-miR-504, hsa-miR-338-5p, hsa-miR-140-3p, hsa-miR-320e, hsa-miR-342-5p, hsa-miR-1207-5p, hsa-miR-4739, hsa-miR-4429, hsa-miR-4484, hsa-miR-320d, hsa-miR-3185, hsa-miR-320c, hsa-miR-320b, hsa-miR-342-3p, hsa-miR-320a, hsa-miR-378b | BRAF, TLN2, GSK3B, PRKCA, PDGFRA, CAPN2, SOS2, ITGB8, CRK, ITGA8, PAK2, COL4A5, RAP1A, ROCK2, BCL2, ITGB5, ARHGAP35,  PTK2, PPP1R12A, CTNNB1, COL6A2,  RELN, COL1A1, ITGA2, PIK3R1, SOS1, RAC1, SRC, PAK6, COL1A2, PRKCB, LAMC1, PDGFD, PTEN, MAPK1, COL5A2, ARHGAP5, MYLK,  PDGFRB, PPP1CB, | 4.2477E-08 | | |
| PI3K-Akt signaling pathway | hsa-miR-503, hsa-miR-133a, hsa-miR-378c, hsa-miR-378d,hsa-miR-422a, hsa-miR-375, hsa-miR-378f, hsa-miR-378i, hsa-miR-1290, hsa-miR-133b, hsa-miR-592, hsa-miR-378e, hsa-miR-1246, hsa-miR-486-5p, hsa-miR-504, hsa-miR-338-5p, hsa-miR-140-3p, hsa-miR-28-3p, hsa-miR-320e, hsa-miR-342-5p, hsa-miR-1207-5p, hsa-miR-4739, hsa-miR-4429, hsa-miR-4484, hsa-miR-320d, hsa-miR-3185, hsa-miR-320c, hsa-miR-320b, hsa-miR-342-3p, hsa-miR-320a, hsa-miR-4309  hsa-miR-378b | PHLPP2, GSK3B, PRKCA, PDGFRA, PPP2R5E, CREB3L3, SOS2, ITGB8, PPP2R3A, ITGA8, COL4A5, YWHAG, MCL1, HSP90AA1, BCL2, YWHAB, PPP2R5C, KRAS, CDK6, RHEB, ITGB5, EFNA5,GHR, IFNAR1,PTK2,BRCA1,JAK2, COL6A2, MLST8, RELN, COL1A1, EIF4B, KIT, ITGA2, PIK3R1, SOS1, YWHAZ, PPP2R3C, KITLG, RAC1,  INSR, COL1A2, LAMC1,  PPP2CB,  BCL2L1,  CREB3L2,  CCNE1, PDGFD, PKN2,  GNG4, PTEN, MAPK1, FGF7, COL5A2, JAK1, PDGFRB, IL6R | 9.6822E-08 | | |
| Fc gamma R-mediated phagocytosis | hsa-miR-4417, hsa-miR-503, hsa-miR-139-5p, hsa-miR-133a, hsa-miR-1290, hsa-miR-378g, hsa-miR-133b, hsa-miR-1246, hsa-miR-486-5p, hsa-miR-504, hsa-miR-338-5p, hsa-miR-140-3p, hsa-miR-28-3p, hsa-miR-320e, hsa-miR-324-3p, hsa-miR-1207-5p, hsa-miR-4739, hsa-miR-4429, hsa-miR-4484, hsa-miR-320d, hsa-miR-320c, hsa-miR-320b, hsa-miR-342-3p  hsa-miR-320a | PRKCA, WASL, INPPL1, CRK, PLD1, PRKCE, PIP5K1B, ASAP1, ASAP3, PPAP2B, MARCKS, DNM1, WASF2, PIK3R1, RAC1, PRKCB, MAPK1, ASAP2 | 0.00050293 | | |
| Pathways in cancer | hsa-miR-215, hsa-miR-503, hsa-miR-133a, hsa-miR-378c, hsa-miR-378d, hsa-miR-422a, hsa-miR-375, hsa-miR-378f, hsa-miR-378i, hsa-miR-1290, hsa-miR-378g  hsa-miR-133b, hsa-miR-592, hsa-miR-378e, hsa-miR-1246, hsa-miR-486-5p, hsa-miR-504, hsa-miR-338-5p  hsa-miR-4485, hsa-miR-140-3p, hsa-miR-28-3p, hsa-miR-320e, hsa-miR-342-5p, hsa-miR-1207-5p, hsa-miR-4739, hsa-miR-4429, hsa-miR-4484, hsa-miR-320d, hsa-miR-3185, hsa-miR-3651, hsa-miR-320c, hsa-miR-320b, hsa-miR-342-3p, hsa-miR-320a, hsa-miR-4309, hsa-miR-378b | BRAF, FOS, GSK3B, PRKCA, DVL3, PDGFRA, WNT16, E2F1, SOS2, CBL, TCF4, APC, CRK, COL4A5, HSP90AA1, FZD6, BCL2, BIRC5, PLD1,  CDKN2B, TRAF5, KRAS, CDK6, TCEB1, PML, EPAS1, MLH1, PTK2, CBLB, SMAD4, CTNNB1, MSH6,  AXIN2, E2F3, EGLN3, KIT, ITGA2, PIK3R1, SOS1HDAC2, KITLG, RAC1, BMP2, PRKCB, MAX, LAMC1, BCL2L1, CCNE1, LEF1, RALB, PTEN, FOXO1, MAPK1, FGF7, JAK1, PDGFRB, EGLN1, RXRA, | 1.3318E-06 | | |
| Bacterial invasion of epithelial cells | hsa-miR-503, hsa-miR-139-5p, hsa-miR-133a, hsa-miR-378c, hsa-miR-378d, hsa-miR-422a, hsa-miR-378f, hsa-miR-378i, hsa-miR-1290, hsa-miR-378g, hsa-miR-133b, hsa-miR-378e, hsa-miR-139-3p, hsa-miR-486-5p, hsa-miR-338-5p, hsa-miR-140-3p, hsa-miR-28-3p, hsa-miR-1207-5p, hsa-miR-4739, hsa-miR-4429, hsa-miR-4484, hsa-miR-320d, hsa-miR-320c, hsa-miR-320b, hsa-miR-320a, hsa-miR-378b, | WASL, CBL, CRK, CLTC, PTK2, CBLB, CTNNB1,  DNM1, WASF2, PIK3R1,  RAC1, SRC, GAB1, | 0.00785347 | | |
| Endocytosis | hsa-miR-4417, hsa-miR-503, hsa-miR-139-5p, hsa-miR-133ª, hsa-miR-378c,hsa-miR-375, hsa-miR-378i, hsa-miR-1290, hsa-miR-133b, hsa-miR-592, hsa-miR-1246, hsa-miR-486-5p, hsa-miR-504, hsa-miR-338-5p  hsa-miR-140-3p, hsa-miR-28-3p, hsa-miR-320e, hsa-miR-324-3p, hsa-miR-1207-5p, hsa-miR-4739, hsa-miR-4429, hsa-miR-4484, hsa-miR-320d, hsa-miR-320c, hsa-miR-320b, hsa-miR-342-3p, hsa-miR-320a, hsa-miR-4309 | PDGFRA, VPS45, CBL, DAB2, AP2B1, SMURF2, NEDD4L, FOLR1, SH3GL1, PLD1, ACAP2, CLTC,  PML, PIP5K1B, ASAP1, ASAP3, VPS4B, CBLB, PSD3, GIT1, VPS37A, SMAP1, DNM1, SMURF1,  CHMP5, RAB11A, KIT, STAM2, SRC, USP8, RAB22A, SMAD7, PARD6B, ADRB1, ARRB1, ASAP2, | 2.2755E-06 | | |
| Regulation of actin cytoskeleton | hsa-miR-215, hsa-miR-503, hsa-miR-139-5p, hsa-miR-133a, hsa-miR-378c, hsa-miR-378d, hsa-miR-422a, hsa-miR-375, hsa-miR-378f, hsa-miR-378i, hsa-miR-1290, hsa-miR-378g, hsa-miR-133b, hsa-miR-592, hsa-miR-378e, hsa-miR-1246, hsa-miR-486-5p, hsa-miR-338-5p, hsa-miR-140-3p, hsa-miR-28-3p, hsa-miR-320e, hsa-miR-342-5p, hsa-miR-1207-5p, hsa-miR-4739, hsa-miR-4429, hsa-miR-4484, hsa-miR-320d, hsa-miR-3185, hsa-miR-320c, hsa-miR-320b, hsa-miR-342-3p, hsa-miR-320a, hsa-miR-4309, hsa-miR-378b | PFN1, BRAF, PDGFRA, WASL, SOS2, ITGB8, APC, CRK, ITGA8, PAK2, ARHGEF12,  GNA13, ROCK2, TIAM1,  KRAS,  RRAS2, ITGB5, PIP5K1B,  ABI2,  ARHGAP35,  PTK2,  PPP1R12A, GIT1,  WASF2, ITGA2,  PIK3R1,  SOS1, RAC1,  SRC,  PAK6,  PDGFD,  MAPK1, FGF7, DIAPH2, MYLK, PDGFRB, PPP1C | 0.00011331 | | |
| ErbB signaling pathway | hsa-miR-215, hsa-miR-503, hsa-miR-133a, hsa-miR-378c, hsa-miR-378d, hsa-miR-422a, hsa-miR-378f, hsa-miR-378i, hsa-miR-1290, hsa-miR-133b, hsa-miR-378e, hsa-miR-1246, hsa-miR-139-3p, hsa-miR-486-5p  hsa-miR-504, hsa-miR-338-5p, hsa-miR-4701-3p, hsa-miR-140-3p, hsa-miR-320e, hsa-miR-1207-5p, hsa-miR-4739, hsa-miR-4429, hsa-miR-4484, hsa-miR-320d, hsa-miR-3185, hsa-miR-320c, hsa-miR-320b, hsa-miR-342-3p, hsa-miR-320a, hsa-miR-378b | CAMK2D, BRAF, GSK3B, PRKCA, SOS2, CBL, CRK,  PAK2, KRAS, PTK2, CBLB, PIK3R1, SOS1, SRC, PAK6, PRKCB, GAB1, MAPK1, ABL2, EREG | 8.1422E-05 | | |
| Cell cycle | hsa-miR-215, hsa-miR-503, hsa-miR-139-5p, hsa-miR-133a, hsa-miR-378c, hsa-miR-378d, hsa-miR-422a, hsa-miR-375, hsa-miR-378f, hsa-miR-378i, hsa-miR-1290, hsa-miR-378g, hsa-miR-133b, hsa-miR-338-5p, hsa-miR-140-3p, hsa-miR-28-3p, hsa-miR-320e, hsa-miR-324-3p, hsa-miR-1207-5p, hsa-miR-4429, hsa-miR-4484, hsa-miR-320d, hsa-miR-3185, hsa-miR-320c, hsa-miR-320b, hsa-miR-342-3p, hsa-miR-320a, hsa-miR-378b | ESPL1, GSK3B, E2F1, ORC2, YWHAG, MCM4, DBF4, FZR1, STAG2, CDKN2B, YWHAB, CDK6, BUB3, CHEK1, TFDP2, SMAD4, E2F3, YWHAZ, HDAC2, CDC7, MAD2L1, CCNE1, CDC27, PRKDC, RAD21, ORC5 | 7.9282E-05 | | |
| B cell receptor signaling pathway | hsa-miR-4417, hsa-miR-503, hsa-miR-133a, hsa-miR-375, hsa-miR-1290, hsa-miR-133b, hsa-miR-486-5p, hsa-miR-338-5p, hsa-miR-140-3p, hsa-miR-28-3p, hsa-miR-320e, hsa-miR-324-3p, hsa-miR-1207-5p, hsa-miR-4739, hsa-miR-4429, hsa-miR-4484, hsa-miR-320d, hsa-miR-3185, hsa-miR-320c, hsa-miR-320b, hsa-miR-320a | FOS, GSK3B, SOS2, INPPL1, BCL10, KRAS, NFATC4, NFAT5, PIK3R1, SOS1, RAC1, PRKCB, NFATC3, MAPK1, | 0.00614909 | | |
| TGF-beta signaling pathway | hsa-miR-503, hsa-miR-139-5p, hsa-miR-133a, hsa-miR-378c, hsa-miR-378d, hsa-miR-422a, hsa-miR-375, hsa-miR-378f, hsa-miR-378i, hsa-miR-1290, hsa-miR-133b, hsa-miR-378e, hsa-miR-1246, hsa-miR-486-5p, hsa-miR-338-5p, hsa-miR-140-3p, hsa-miR-320e, hsa-miR-342-5p, hsa-miR-1207-5p, hsa-miR-4739, hsa-miR-4429, hsa-miR-4484, hsa-miR-320d, hsa-miR-320c, hsa-miR-320b, hsa-miR-342-3p, hsa-miR-320a, hsa-miR-378b | ID2, INHBB, SMURF2, ROCK2, INHBA, CDKN2B,  ACVR2B, SMAD4, SMURF1, ACVR2A, BMP2, PPP2CB, BMPR1A, SMAD7, BMP7, MAPK1 | 0.00050164 | | |
| VEGF signaling pathway | hsa-miR-4417, hsa-miR-503, hsa-miR-133a, hsa-miR-378c, hsa-miR-378d, hsa-miR-422a, hsa-miR-378f, hsa-miR-378i, hsa-miR-1290, hsa-miR-133b, hsa-miR-378e, hsa-miR-486-5p, hsa-miR-504, hsa-miR-338-5p, hsa-miR-140-3p, hsa-miR-320e, hsa-miR-1207-5p, hsa-miR-4739, hsa-miR-4429, hsa-miR-4484, hsa-miR-320d, hsa-miR-320c, hsa-miR-320b, hsa-miR-320a, hsa-miR-378b | PRKCA, KRAS, NFATC4, PTK2, NFAT5, PIK3R1, RAC1, SRC, PRKCB, NFATC3, MAPK1 | 0.003776178 | | |
| p53 signaling pathway | hsa-miR-503, hsa-miR-133a, hsa-miR-422a, hsa-miR-133b, hsa-miR-592, hsa-miR-378e, hsa-miR-486-5p, hsa-miR-504, hsa-miR-338-5p, hsa-miR-320e, hsa-miR-1207-5p, hsa-miR-4739, hsa-miR-4429, hsa-miR-4484, hsa-miR-320d, hsa-miR-320c, hsa-miR-320b, hsa-miR-342-3p, hsa-miR-320a | CDK6, CHEK1, PMAIP1, SHISA5, EI24, RCHY1, SIAH1, SERPINB5, CCNE1, RRM2, PTEN, SERPINE1, CCNG2, PPM1D | 0.00096976 | | |
