# Supplementary material for: Genomic characterization of liver metastases from colorectal cancer patients
Source: Oncotarget. 2016 Sep 20;7(45):72908–22. doi: 10.18632/oncotarget.12140 (PMC5341953; doi:10.18632/oncotarget.12140)
Supplement: Supplementary file 4 [file oncotarget-07-72908-s004.docx]

**SUPPLEMENTARY TABLE 4.** Most representative canonical pathways and their corresponding genes involved in colorectal liver metastases (n=19) as identified through analysis of the GEP of coding and non-coding RNAs (FDR < .01).

| **Canonical Pathways** | **miRNAs ID** | **Gene ID** | **FDR** |
| --- | --- | --- | --- |
| Focal adhesion | hsa-miR-133a, hsa-miR-1290, hsa-miR-133b, hsa-miR-503, hsa-miR-885-5p, hsa-miR-1246, hsa-miR-592, hsa-miR-3663-3p, hsa-miR-215, hsa-miR-1, hsa-miR-375, hsa-miR-378c, hsa-miR-378d, hsa-miR-4793-3p, hsa-miR-4440, hsa-miR-486-5p, hsa-miR-378i, hsa-miR-378f, hsa-miR-422a, hsa-miR-378g, hsa-miR-483-5p, hsa-miR-4484, hsa-miR-3185, hsa-miR-378e, hsa-miR-504, hsa-miR-1231, hsa-miR-1910, hsa-miR-3175  hsa-miR-3135b, hsa-miR-4739, hsa-miR-3188, hsa-miR-4665-5p, hsa-miR-4741, hsa-miR-3162-5p, hsa-miR-1275, hsa-miR-2392, hsa-miR-4505, hsa-miR-4758-5p, hsa-miR-1202, hsa-miR-146b-5p, hsa-miR-181d, hsa-miR-320d, hsa-miR-421, hsa-miR-939, hsa-miR-4322, hsa-miR-3679-5p, hsa-miR-140-3p, hsa-miR-4443, hsa-miR-4429, hsa-miR-885-3p, hsa-miR-501-5p, hsa-miR-4492, hsa-miR-4695-5p, hsa-miR-4689, hsa-miR-4253, hsa-miR-4763-3p, hsa-miR-28-5p  hsa-miR-320e, hsa-miR-1972, hsa-miR-762, hsa-miR-3665, hsa-miR-4534, hsa-miR-4463, hsa-miR-4656, hsa-miR-3617, hsa-miR-320b, hsa-miR-320c, hsa-miR-320a, hsa-miR-1273f, hsa-miR-1207-5p, hsa-miR-152  hsa-miR-4728-5p, hsa-miR-4687-3p, hsa-miR-4299, hsa-miR-4516, hsa-miR-342-5p, hsa-miR-4710, hsa-miR-342-3p, hsa-miR-3609, hsa-miR-1827, hsa-miR-4510, hsa-let-7c | BRAF, ACTB, TLN2, GSK3B, PRKCA, PDGFRA, CAPN2, ROCK1, SOS2, CRK, SHC1, THBS1, ITGA8, PAK2, CAV1, COL4A5, PPP1CC, RAP1A, CCND2, COL27A1, ITGB4, LAMA1, BCL2, IGF1R, VCL, RHOA, VAV2, AKT2, ARHGAP35, PTK2, LAMC3, PPP1R12A, JUN, COL6A2, PARVA,  DIAPH1, COL5A1, COL1A1, PIK3R1, SOS1, RAC1,  SRC,  CDC42, PAK6, COL1A2, PRKCB,  LAMC1,  IGF1, ITGA7, COL11A1, PDGFD, PIK3CA, FN1, ITGA4, COMP, LAMC2, PTEN, MAPK1, TLN1,  COL5A2, MYL12A, SPP1, ARHGAP5, EGFR, MYLK, MAPK10, PPP1CB, LAMA4 | 6.52688E-11 |
| PI3K-Akt signaling pathway | hsa-miR-133a, hsa-miR-4417, hsa-miR-1290, hsa-miR-133b, hsa-miR-503, hsa-miR-885-5p, hsa-miR-1246, hsa-miR-592, hsa-miR-3663-3p, hsa-miR-1, hsa-miR-375, hsa-miR-378c, hsa-miR-378d, hsa-miR-4793-3p, hsa-miR-4440, hsa-miR-486-5p, hsa-miR-378i, hsa-miR-378f, hsa-miR-422a, hsa-miR-378g, hsa-miR-483-5p, hsa-miR-4484, hsa-miR-3185, hsa-miR-378e, hsa-miR-4449, hsa-miR-504, hsa-miR-1231, hsa-miR-1910, hsa-miR-4745-5p, hsa-miR-3175, hsa-miR-4674, hsa-miR-4651, hsa-miR-3135b, hsa-miR-4739, hsa-miR-3188, hsa-miR-4665-5p, hsa-miR-4741, hsa-miR-3162-5p, hsa-miR-1275, hsa-miR-2392, hsa-miR-4505, hsa-miR-4758-5p, hsa-miR-1908, hsa-miR-1202, hsa-miR-146b-5p, hsa-miR-181d, hsa-miR-320d, hsa-miR-4281  hsa-miR-421, hsa-miR-4721, hsa-miR-939, hsa-miR-4322, hsa-miR-1224-5p, hsa-miR-3679-5p, hsa-miR-140-3p, hsa-miR-4443, hsa-miR-4429, hsa-miR-885-3p  hsa-miR-501-5p, hsa-miR-4492, hsa-miR-4695-5p, hsa-miR-4689, hsa-miR-4253, hsa-miR-4763-3p, hsa-miR-28-5p, hsa-miR-320e, hsa-miR-1972, hsa-miR-762, hsa-miR-3665, hsa-miR-4534, hsa-miR-4463, hsa-miR-1587, hsa-miR-4656, hsa-miR-3617, hsa-miR-320b, hsa-miR-320c, hsa-miR-320a, hsa-miR-1273f, hsa-miR-1207-5p, hsa-miR-4488, hsa-miR-152, hsa-miR-4728-5p, hsa-miR-455-3p, hsa-miR-4687-3p, hsa-miR-542-5p, hsa-miR-4299, hsa-miR-4516, hsa-miR-3198, hsa-miR-342-5p, hsa-miR-4710, hsa-miR-4309, hsa-miR-342-3p, hsa-miR-3609, hsa-miR-1827, hsa-miR-4510, hsa-let-7c, | PHLPP2, PRLR, GSK3B, PRKCA, RBL2,  PDGFRA, NFKB1, PPP2R5E, SOS2, PPP2R3A, THBS1, ITGA8, COL4A5, CDC37, MCL1, CDK2, CCND2, COL27A1, HSP90AA1, ANGPT2, STK11, ITGB4, LAMA1, BCL2, CDKN1B, EFNA3, EIF4EBP1, IGF1R, GNB1, PPP2R5C, KRAS, CDK6, EFNA5, RPS6KB2, PPP2R5A, TP53, IFNAR1, IKBKB, AKT2, PTK2, LAMC3, G6PC, BRCA1, THEM4, CRTC2, DDIT4, JAK2, EIF4E, LPAR1, MYC, COL6A2, NOS3, MLST8, COL5A1, CSF3R, COL1A1, GNG7,  EIF4B, GNG2, PIK3R1, SOS1, YWHAZ, PPP2R3C, KITLG, RAC1, FGF2, COL1A2, LAMC1, IGF1, ITGA7, PPP2CB, BCL2L1, COL11A1, EIF4E2, CREB3L2, CCNE1, PDGFD, PIK3CA, FN1, PKN2, CDKN1A, GNG4, ITGA4, COMP, LAMC2, MTOR, FGFR2, RELA, PTEN, FGFR1, MAPK1, PPP2R1B, FGF7, COL5A2, SPP1, TEK, SGK1, JAK1, EGF, IL6R, LAMA4 | 2.23985E-09 |
| Pathways in cancer | hsa-miR-133a, hsa-miR-4417, hsa-miR-1290, hsa-miR-133b, hsa-miR-503, hsa-miR-885-5p, hsa-miR-1246, hsa-miR-592, hsa-miR-3663-3p, hsa-miR-215, hsa-miR-1, hsa-miR-3180-3p, hsa-miR-375, hsa-miR-378c hsa-miR-378d, hsa-miR-4793-3p, hsa-miR-4440, hsa-miR-486-5p, hsa-miR-378i, hsa-miR-378f, hsa-miR-422a, hsa-miR-378g, hsa-miR-4484, hsa-miR-3185, hsa-miR-378e, hsa-miR-3180, hsa-miR-504, hsa-miR-1231, hsa-miR-1910, hsa-miR-4745-5p, hsa-miR-4674, hsa-miR-4651, hsa-miR-4649-5p, hsa-miR-3135b, hsa-miR-4739, hsa-miR-3188, hsa-miR-4665-5p, hsa-miR-4741, hsa-miR-3162-5p, hsa-miR-1275, hsa-miR-2392, hsa-miR-1225-5p, hsa-miR-3656, hsa-miR-4505, hsa-miR-4758-5p, hsa-miR-3195, hsa-miR-1202, hsa-miR-146b-5p, hsa-miR-181d, hsa-miR-320d, hsa-miR-421, hsa-miR-4721, hsa-miR-939, hsa-miR-4322, hsa-miR-1224-5p, hsa-miR-3679-5p, hsa-miR-140-3p, hsa-miR-28-3p, hsa-miR-4443, hsa-miR-4429, hsa-miR-885-3p, hsa-miR-501-5p, hsa-miR-4492, hsa-miR-4695-5p, hsa-miR-4689, hsa-miR-4763-3p, hsa-miR-28-5p, hsa-miR-320e, hsa-miR-1972, hsa-miR-762, hsa-miR-3665, hsa-miR-4534, hsa-miR-4463, hsa-miR-1587, hsa-miR-4656, hsa-miR-3617, hsa-miR-320b, hsa-miR-320c, hsa-miR-320a, hsa-miR-1273f, hsa-miR-1207-5p, hsa-miR-152, hsa-miR-4728-5p, hsa-miR-455-3p, hsa-miR-4687-3p, hsa-miR-542-5p, hsa-miR-4299, hsa-miR-4516, hsa-miR-342-5p, hsa-miR-4710, hsa-miR-4309, hsa-miR-342-3p, hsa-miR-3609, hsa-miR-1827, hsa-miR-4510, hsa-let-7c | BRAF, FOS, GSK3B, PRKCA, DVL3, PDGFRA, WNT16, E2F1, NFKB1, WNT7A, SOS2, SMAD2, CBL, BID, TCF4, APC, CRK, WNT5A, CTBP1, COL4A5, ZBTB16, CDK2, HSP90AA1, RALA, LAMA1, BCL2, CDKN1B, WNT2B, TRAF4, BIRC5, RALBP1, CDKN2B, IGF1R, RHOA, TRAF5, WNT5B, FZD8, KRAS, CDK6, TCEB1, MST1, PML, EPAS1, MLH1, TP53, MITF, IKBKB, AKT2, PTK2, LAMC3, CBLB, RUNX1T1, ARNT2, CASP3, JUN, SMAD4, AXIN2, E2F3, MYC, NFKB2, GLI3, CSF3R, TRAF2, PIK3R1, SOS1, HDAC2, KITLG, NOS2, RAC1, FGF2, CDC42, RARA, BMP2, PRKCB, MAX, FAS, LAMC1, IGF1, EP300, BCL2L1, CCNE1, PIK3CA, LEF1, FN1, CDKN1A, RALB, SLC2A1, LAMC2, STAT1, CYCS, MTOR, FGFR2, RELA, RXRB, TCF7, PTEN, FGFR1, FOXO1, MAPK1, FGF7, TRAF1, PPARD, JAK1, EGF, STK36, MAPK10, EGLN1, LAMA4 | 4.98493E-10 |
| Bacterial invasion of epithelial cells | hsa-miR-133a, hsa-miR-1290, hsa-miR-133b, hsa-miR-503, hsa-miR-885-5p, hsa-miR-592, hsa-miR-3663-3p, hsa-miR-1, hsa-miR-378c, hsa-miR-378d, hsa-miR-486-5p, hsa-miR-378i, hsa-miR-378f, hsa-miR-422a, hsa-miR-378g, hsa-miR-139-5p, hsa-miR-4484, hsa-miR-378e, hsa-miR-3175, hsa-miR-4739, hsa-miR-3188, hsa-miR-4665-5p, hsa-miR-1275, hsa-miR-2392,  hsa-miR-139-3p, hsa-miR-146b-5p, hsa-miR-181d, hsa-miR-320d, hsa-miR-4721, hsa-miR-3679-5p, hsa-miR-140-3p, hsa-miR-28-3p, hsa-miR-4443, hsa-miR-4429, hsa-miR-885-3p, hsa-miR-501-5p, hsa-miR-4492, hsa-miR-4695-5p, hsa-miR-4689, hsa-miR-4763-3p, hsa-miR-762, hsa-miR-4534, hsa-miR-3617, hsa-miR-320b, hsa-miR-320c, hsa-miR-320a, hsa-miR-1207-5p, hsa-miR-152, hsa-miR-4728-5p, hsa-miR-4687-3p, hsa-miR-342-5p, hsa-miR-4710, hsa-miR-4309, hsa-miR-1827, hsa-miR-4510, hsa-let-7c | ARPC5, ACTB, WASL, CBL, CRK, SHC1, CAV1, CLTC, VCL, RHOA, PTK2, CBLB, DNM1, WASF2, PIK3R1, RAC1, SRC, CDC42, GAB1, PIK3CA, FN1, ARPC4, CLTB | 0.001055145 |
| Endocytosis | hsa-miR-133a, hsa-miR-4417, hsa-miR-1290, hsa-miR-133b, hsa-miR-503, hsa-miR-885-5p, hsa-miR-1246, hsa-miR-592, hsa-miR-3663-3p, hsa-miR-1, hsa-miR-3180-3p, hsa-miR-375, hsa-miR-378c, hsa-miR-378d, hsa-miR-4793-3p, hsa-miR-486-5p, hsa-miR-378i, hsa-miR-378f, hsa-miR-422a, hsa-miR-4486, hsa-miR-483-5p, hsa-miR-139-5p, hsa-miR-4484, hsa-miR-3185, hsa-miR-378e, hsa-miR-3180, hsa-miR-504, hsa-miR-1231, hsa-miR-4745-5p, hsa-miR-3175, hsa-miR-4674, hsa-miR-4651, hsa-miR-4649-5p, hsa-miR-3135b, hsa-miR-4739, hsa-miR-3188, hsa-miR-4665-5p, hsa-miR-1275, hsa-miR-4505, hsa-miR-4758-5p, hsa-miR-1202, hsa-miR-146b-5p, hsa-miR-320d, hsa-miR-3679-5p, hsa-miR-140-3p, hsa-miR-4429, hsa-miR-885-3p, hsa-miR-501-5p, hsa-miR-663b, hsa-miR-4492, hsa-miR-4695-5p, hsa-miR-4689, hsa-miR-4763-3p, hsa-miR-28-5p, hsa-miR-320e, hsa-miR-762, hsa-miR-1587, hsa-miR-4656, hsa-miR-3617, hsa-miR-320b, hsa-miR-320c, hsa-miR-320a, hsa-miR-1273f, hsa-miR-1207-5p, hsa-miR-152, hsa-miR-574-3p, hsa-miR-4728-5p, hsa-miR-455-3p, hsa-miR-4687-3p, hsa-miR-4299, hsa-miR-4516, hsa-miR-3198, hsa-miR-4710, hsa-miR-4309, hsa-miR-342-3p, hsa-miR-3609, hsa-miR-4510, hsa-let-7c | PDGFRA, VPS45, ADRBK1, SMAD2, SMAD6, CBL, VPS37D, GRK5, VTA1, WWP1, DAB2, PDCD6IP, CAV1, SMURF2, NEDD4L, FOLR1, SH3GL1, AGAP1, ADRB2, ACAP2, IGF1R, CLTC, RHOA, ERBB3, PML, PIP5K1B, ARFGAP1, TSG101, ASAP1, RAB5A, ASAP3, CBLB, PSD3, CHMP2B, GIT1, GIT2, VPS37A, SMAP1, EPS15, DNM1, SMURF1, CHMP5, ZFYVE9, RAB11A, STAM2, SRC, CDC42, AP2A1, USP8, GRK6, ZFYVE20, RAB11B, SMAD7, FGFR2, CLTB, PARD6B, ADRB1, EGF, ARRB1 | 0.000542512 |
| Regulation of actin cytoskeleton | hsa-miR-133a, hsa-miR-1290, hsa-miR-133b, hsa-miR-503, hsa-miR-885-5p, hsa-miR-1246, hsa-miR-592, hsa-miR-3663-3p, hsa-miR-215, hsa-miR-1, hsa-miR-375, hsa-miR-3178, hsa-miR-378c, hsa-miR-378d, hsa-miR-4793-3p, hsa-miR-486-5p, hsa-miR-378i, hsa-miR-378f, hsa-miR-422a, hsa-miR-378g, hsa-miR-483-5p, hsa-miR-139-5p, hsa-miR-4484, hsa-miR-3185, hsa-miR-378e, hsa-miR-1231, hsa-miR-1910, hsa-miR-4745-5p, hsa-miR-3175, hsa-miR-4674, hsa-miR-4651,  hsa-miR-3135b, hsa-miR-4739, hsa-miR-3188, hsa-miR-4665-5p, hsa-miR-4741, hsa-miR-3162-5p, hsa-miR-1275, hsa-miR-2392, hsa-miR-4505, hsa-miR-4758-5p, hsa-miR-1202, hsa-miR-146b-5p, hsa-miR-181d, hsa-miR-320d, hsa-miR-421, hsa-miR-4721, hsa-miR-939, hsa-miR-4322, hsa-miR-1224-5p, hsa-miR-3679-5p, hsa-miR-140-3p, hsa-miR-28-3p, hsa-miR-4443, hsa-miR-4429, hsa-miR-885-3p, hsa-miR-501-5p, hsa-miR-4492, hsa-miR-4695-5p, hsa-miR-4689, hsa-miR-4253, hsa-miR-4763-3p, hsa-miR-28-5p, hsa-miR-320e, hsa-miR-1972, hsa-miR-762, hsa-miR-3665, hsa-miR-4534, hsa-miR-4463, hsa-miR-1587, hsa-miR-4656, hsa-miR-3617, hsa-miR-320b, hsa-miR-320c, hsa-miR-320a, hsa-miR-1273f, hsa-miR-1207-5p, hsa-miR-152, hsa-miR-4728-5p, hsa-miR-455-3p, hsa-miR-4687-3p, hsa-miR-4299, hsa-miR-4516, hsa-miR-342-5p, hsa-miR-4710, hsa-miR-4309, hsa-miR-342-3p, hsa-miR-3609, hsa-miR-1827,  hsa-miR-4510, hsa-let-7c | ARPC5, BRK1, PFN1, BRAF, ACTB, PDGFRA, CYFIP2, ROCK1, WASL, ITGAX, SOS2, BAIAP2, APC, CRK, IQGAP3, ITGA8, PAK2, ARHGEF12, PPP1CC, GNA13, IQGAP1, ITGB4, TIAM1, VCL, RHOA, KRAS, RRAS2, PIP5K1B, ABI2, ARHGEF1, VAV2, ARHGAP35, PTK2, SLC9A1, PIKFYVE, PPP1R12A, GIT1, IQGAP2, DIAPH1, WASF2, CFL2, PIK3R1, SOS1, RAC1, SRC, FGF2, CDC42, PAK6, ITGA7, PDGFD, PIK3CA, LIMK1, FN1, ITGA4, ARPC4, ARHGEF7, NCKAP1, FGFR2, FGFR1, MAPK1, FGF7, DIAPH2, MYL12A, EGF, MYLK, PPP1CB | 7.81056E-07 |
| ErbB signaling pathway | hsa-miR-133a, hsa-miR-1290, hsa-miR-133b, hsa-miR-503, hsa-miR-885-5p, hsa-miR-1246, hsa-miR-592, hsa-miR-3663-3p, hsa-miR-215, hsa-miR-1, hsa-miR-378c, hsa-miR-378d, hsa-miR-4793-3p, hsa-miR-4440, hsa-miR-486-5p, hsa-miR-378i, hsa-miR-378f, hsa-miR-422a, hsa-miR-4486, hsa-miR-4484, hsa-miR-3185, hsa-miR-378e, hsa-miR-4449, hsa-miR-504, hsa-miR-1231, hsa-miR-1910, hsa-miR-4649-5p, hsa-miR-4739, hsa-miR-4665-5p, hsa-miR-4741, hsa-miR-1275, hsa-miR-4505, hsa-miR-4758-5p, hsa-miR-1202, hsa-miR-139-3p, hsa-miR-146b-5p, hsa-miR-181d, hsa-miR-320d, hsa-miR-4281, hsa-miR-421, hsa-miR-939, hsa-miR-4322, hsa-miR-3679-5p, hsa-miR-140-3p, hsa-miR-4443, hsa-miR-4429, hsa-miR-885-3p, hsa-miR-501-5p, hsa-miR-4492, hsa-miR-4695-5p, hsa-miR-4689, hsa-miR-4763-3p, hsa-miR-28-5p, hsa-miR-320e, hsa-miR-1972, hsa-miR-762, hsa-miR-3665, hsa-miR-4463, hsa-miR-1587, hsa-miR-4656, hsa-miR-3617, hsa-miR-320b, hsa-miR-320c, hsa-miR-320a, hsa-miR-1273f, hsa-miR-1207-5p, hsa-miR-152, hsa-miR-4728-5p, hsa-miR-4687-3p, hsa-miR-4299, hsa-miR-4516, hsa-miR-342-5p, hsa-miR-4710, hsa-miR-342-3p, hsa-miR-3609, hsa-miR-1827, hsa-miR-4510, hsa-let-7c | CAMK2D, BRAF, GSK3B, PRKCA, SOS2, CBL, CAMK2G, CRK, SHC1, PAK2, MAP2K7, CDKN1B, EIF4EBP1, ERBB3, KRAS, RPS6KB2, NCK1, AKT2, PTK2, CBLB, JUN, MYC, PIK3R1, SOS1, SRC, PAK6, BTC, PRKCB, GAB1, PIK3CA, CDKN1A, MTOR, MAP2K4, MAPK1, ABL2, EGF, MAPK10, EREG | 6.52688E-11 |
| B cell receptor signaling pathway | hsa-miR-133a, hsa-miR-4417, hsa-miR-1290, hsa-miR-133b, hsa-miR-503, hsa-miR-592, hsa-miR-3663-3p, hsa-miR-1, hsa-miR-375, hsa-miR-4793-3p, hsa-miR-4440, hsa-miR-486-5p, hsa-miR-4484, hsa-miR-3185, hsa-miR-1231, hsa-miR-1910, hsa-miR-4745-5p, hsa-miR-3135b, hsa-miR-4739, hsa-miR-3188, hsa-miR-4741, hsa-miR-2392, hsa-miR-4758-5p, hsa-miR-1202, hsa-miR-146b-5p, hsa-miR-181d, hsa-miR-320d, hsa-miR-4322, hsa-miR-1224-5p, hsa-miR-3679-5p, hsa-miR-140-3p, hsa-miR-28-3p, hsa-miR-4443, hsa-miR-4429, hsa-miR-885-3p, hsa-miR-501-5p, hsa-miR-4492, hsa-miR-4695-5p, hsa-miR-4689, hsa-miR-28-5p, hsa-miR-320e, hsa-miR-762, hsa-miR-4534, hsa-miR-4463, hsa-miR-1587, hsa-miR-4656, hsa-miR-3617, hsa-miR-320b, hsa-miR-320c, hsa-miR-320a, hsa-miR-1207-5p, hsa-miR-152, hsa-miR-4728-5p, hsa-miR-542-5p, hsa-miR-4516, hsa-miR-342-5p, hsa-miR-4710, hsa-miR-3609, hsa-miR-1827, hsa-miR-4510, hsa-let-7c | FOS, GSK3B, NFKB1, SOS2, BCL10, KRAS, NFATC4, VAV2, IKBKB, PPP3CA, AKT2, NFAT5, JUN, PPP3CB, BTK, PIK3R1, SOS1, RAC1, PRKCB, PIK3CA, RELA, NFATC3, MAPK1 | 0.006045669 |
| TGF-beta signaling pathway | hsa-miR-133a, hsa-miR-1290, hsa-miR-133b, hsa-miR-503, hsa-miR-1246, hsa-miR-592, hsa-miR-3663-3p, hsa-miR-1, hsa-miR-375, hsa-miR-378c, hsa-miR-378d, hsa-miR-4793-3p, hsa-miR-486-5p, hsa-miR-378i, hsa-miR-378f, hsa-miR-422a, hsa-miR-378g, hsa-miR-139-5p, hsa-miR-4484, hsa-miR-378e, hsa-miR-4449, hsa-miR-1231, hsa-miR-1910, hsa-miR-4651, hsa-miR-4739, hsa-miR-3188, hsa-miR-4741, hsa-miR-3162-5p, hsa-miR-1275, hsa-miR-2392, hsa-miR-4505, hsa-miR-1202, hsa-miR-146b-5p, hsa-miR-320d, hsa-miR-4281, hsa-miR-421, hsa-miR-939, hsa-miR-1224-5p, hsa-miR-140-3p, hsa-miR-4443, hsa-miR-4429, hsa-miR-501-5p, hsa-miR-4492, hsa-miR-4695-5p, hsa-miR-4689, hsa-miR-320e, hsa-miR-762, hsa-miR-3665, hsa-miR-4534, hsa-miR-4463, hsa-miR-1587, hsa-miR-3617, hsa-miR-320b, hsa-miR-320c, hsa-miR-320a, hsa-miR-1273f, hsa-miR-1207-5p, hsa-miR-152, hsa-miR-4728-5p, hsa-miR-455-3p, hsa-miR-4687-3p, hsa-miR-3198, hsa-miR-4309, hsa-miR-342-3p, hsa-miR-1827, hsa-miR-4510, hsa-let-7c | ID, ROCK1, SMAD2, SMAD6, INHBB, THBS1, SMURF2, CHRD, INHBA, CDKN2B, RHOA, SKP1, RPS6KB2, ACVR2B, SMAD4, E2F5, MYC, SMURF1, ZFYVE9, SMAD5, ACVR2A, BMP2, SP1, EP300, PPP2CB, BMPR1A, COMP, SMAD7, E2F4, TNF, BMP7, MAPK1, PPP2R1B, INHBE, BMPR2 | 2.06528E-13 |
| p53 signaling pathway | hsa-miR-133a, hsa-miR-1290, hsa-miR-133b, hsa-miR-503, hsa-miR-885-5p, hsa-miR-592, hsa-miR-3663-3p,  hsa-miR-1, hsa-miR-4793-3p, hsa-miR-486-5p, hsa-miR-422a, hsa-miR-4484, hsa-miR-3185, hsa-miR-378e, hsa-miR-504, hsa-miR-3135b, hsa-miR-4739, hsa-miR-2392, hsa-miR-4505, hsa-miR-4758-5p, hsa-miR-1202, hsa-miR-181d, hsa-miR-320d, hsa-miR-421, hsa-miR-939, hsa-miR-1224-5p, hsa-miR-4443, hsa-miR-4429, hsa-miR-885-3p, hsa-miR-4695-5p, hsa-miR-4689, hsa-miR-4763-3p, hsa-miR-28-5p, hsa-miR-320e, hsa-miR-3617, hsa-miR-320b, hsa-miR-320c, hsa-miR-320a, hsa-miR-1273f, hsa-miR-1207-5p, hsa-miR-152, hsa-miR-4728-5p, hsa-miR-4687-3p, hsa-miR-4299, hsa-miR-4710, hsa-miR-3609, hsa-miR-1827, hsa-let-7c, | CCNG1, BID, THBS1, CDK2, CCND2, CDK6, CHEK1, TP53, PMAIP1, CASP3, SHISA5, RCHY1, SESN1, SESN2, MDM4, FAS, IGF1, SERPINB5, CCNE1, CDKN1A, CYCS, STEAP3, PTEN, SERPINE1, CCNG2, PPM1D | 7.25729E-06 |
